# Isolation, identification and comparative genomic analysis of *Lactobacillus salivarius* from Mongolian horse vagina

**DOI:** 10.3389/fmicb.2025.1635639

**Published:** 2025-07-31

**Authors:** Yiping Zhao, Yuanyi Liu, Jinshan Tao, Jialong Cao, Yanan Lin, Qianqian He, Xinlan Fang, Siqin Yun, Ming Du, Shaofeng Su, Tugeqin Bao, Dongyi Bai, Xinzhuang Zhang, Manglai Dugarjaviin

**Affiliations:** ^1^Key Laboratory of Equus Germplasm Innovation, Ministry of Agriculture and Rural Affairs, Hohhot, China; ^2^Inner Mongolia Key Laboratory of Equine Science Research and Technology Innovation, Inner Mongolia Agricultural University, Hohhot, China; ^3^College of Animal Science, Inner Mongolia Agricultural University, Hohhot, China; ^4^Inner Mongolia Academy of Agricultural & Animal Husbandry Sciences, Hohhot, China

**Keywords:** *Lactobillus salivarius*, probiotics, vaginal microbiota, comparative genomics, Mongolian horse

## Abstract

**Introduction:**

Reproductive health in mares is pivotal for the sustainability of the equine industry, yet vaginal microbiota dysbiosis remains an underrecognized contributor to infections such as endometritis and bacterial vaginosis. While *Lactobacillus* spp. dominate healthy vaginal ecosystems in humans and livestock, their role in equine reproductive health, particularly in resilient breeds like Mongolian mares, is poorly understood. This study aimed to isolate and characterize a novel *Lactobacillus* strain from the vaginal microbiota of healthy Mongolian mares and evaluate its probiotic potential for mitigating equine reproductive disorders.

**Methods:**

A polyphasic approach integrating phenotypic, biochemical, and 16S rRNA gene sequencing was employed to isolate and identify a novel *Lactobacillus salivarius* strain (Y20) from the vaginal microbiota of healthy Mongolian mares. The probiotic potential of Y20 was assessed through *in vitro* assays, including tolerance to low pH (pH 2.5) and bile salts (0.3%), antagonistic activity against equine pathogens (*Staphylococcus aureus*, *Escherichia coli*, *Pseudomonas aeruginosa*), and antioxidant capacity (DPPH radical scavenging assay). Whole-genome sequencing (1.74 Mb, 33.01% GC content) was performed to analyze genes related to carbohydrate metabolism, adhesion factors, bacteriocin biosynthesis, and stress response pathways. Comparative genomics was used to explore phylogenetic relationships and genomic adaptations for vaginal colonization.

**Results:**

*Lactobacillus salivarius* Y20 demonstrated robust tolerance to low pH and bile salts, potent antagonistic activity against key equine pathogens, and significant antioxidant capacity (82.4% DPPH radical scavenging). Genomic analysis revealed genes encoding carbohydrate metabolism, adhesion factors, bacteriocin biosynthesis (including a novel putative bacteriocin cluster), and stress response pathways. Comparative genomics confirmed Y20’s close phylogenetic relationship with horse-derived *Lactobacillus salivarius* strains and identified unique genomic adaptations for vaginal colonization.

**Discussion:**

These findings identify *Lactobacillus salivarius* Y20 as a promising candidate probiotic for mitigating equine reproductive disorders. Its multifaceted probiotic properties, including pathogen inhibition, antioxidant activity, and genomic adaptations for vaginal colonization, suggest potential applications in sustainable equine health management. This study advances microbiome-based strategies as viable alternatives to antibiotics, offering new avenues for improving reproductive health in mares.

## 1 Introduction

The equine industry, a vital component of global agriculture, recreation, and cultural heritage, hinges on the reproductive health of mares to sustain productivity, genetic diversity, and economic viability. However, reproductive tract infections (RTIs) in mares remain a persistent and costly challenge, with far-reaching consequences for equine welfare and breeding programs. RTIs are frequently linked to dysbiosis of the vaginal microbiota-a complex ecosystem that, when disrupted, can precipitate adverse outcomes such as infertility, recurrent abortion, chronic endometritis, and premature ovarian failure (Edwards, 2008; Sharifzadeh and Shokri, 2021). These conditions reduce foaling rates, increase veterinary costs and culling risks, and necessitate reliance on assisted reproductive technologies, threatening the long-term sustainability of equine operations.

The vaginal microbiota of healthy mares acts as a dynamic, species-specific barrier against pathogens. However, its composition and functional role are less understood compared to those in humans and livestock (Gil-Miranda et al., 2024). Studies in humans and rodents have underscored the critical importance of *Lactobacilli*-dominated microbiota in maintaining vaginal pH homeostasis, producing antimicrobial compounds (e.g., lactic acid, hydrogen peroxide, bacteriocins), and competing with pathogens for adhesion sites (Kovachev, 2018; Malaluang et al., 2024). In contrast, equine vaginal microbiota research has historically prioritized pathogenic microorganisms-such as *Staphylococcus aureus*, *Escherichia coli*, *Pseudomonas aeruginosa*, and *Streptococcus zooepidemicus*-which are frequently implicated in endometritis, pyometra, and neonatal sepsis (Barba et al., 2020; da Silva-Álvarez et al., 2025). While this pathogen-centric approach has advanced diagnostic and therapeutic strategies, it has inadvertently overshadowed investigations into the role of commensal bacteria in conferring resilience against RTIs.

Among *Lactobacilli*, *Lactobacillus salivarius* stands out as a model probiotic with documented benefits in humans, poultry, and ruminants. In humans, *Lactobacillus salivarius* strains have been shown to mitigate urogenital infections by modulating immune responses, producing broad-spectrum bacteriocins (e.g., salivaricin A/B), and enhancing epithelial barrier integrity (Messaoudi et al., 2013; Wang et al., 2022). Similarly, in livestock, *Lactobacillus salivarius* has demonstrated efficacy in preventing gastrointestinal disorders, reducing pathogen colonization, and improving weight gain (Yang et al., 2023; Yang et al., 2024). Despite these parallels, the role of *Lactobacillus salivarius* in the equine vaginal ecosystem remains largely unexplored, particularly in indigenous breeds adapted to extreme environments.

Mongolian horses, renowned for their endurance, cold tolerance, and foraging adaptability, represent a unique genetic resource for studying host-microbiome interactions under harsh conditions (Wen et al., 2022). Their reproductive physiology, shaped by millennia of natural selection in the steppe ecosystem, may favor microbiota profiles distinct from those of domesticated breeds raised in controlled environments. However, modernization of equine husbandry-including intensified breeding practices, antibiotic overuse, and reduced genetic diversity-has introduced novel stressors that could destabilize the vaginal microbiota. For instance, artificial insemination, while increasing breeding efficiency, may disrupt microbial transmission and expose the reproductive tract to exogenous pathogens (Cazales et al., 2020). Similarly, antibiotic therapies, while critical for treating RTIs, can inadvertently erode protective microbiota, fostering antibiotic resistance and recurrent infections (de la Fuente-Nunez et al., 2023).

Probiotic interventions offer a sustainable alternative to antibiotics by restoring microbial balance and enhancing host defenses. In equine medicine, probiotics have shown promise in preventing diarrhea, modulating gut-brain axis signaling, and reducing post-surgical complications (Lai et al., 2023; Boucher et al., 2024). However, their application to the vaginal microbiota is nascent, with limited studies investigating strain-specific traits (e.g., acid/bile tolerance, adhesion, antimicrobial activity) essential for colonization and efficacy. For instance, specific lactic acid bacteria combinations have been shown to regulate the vaginal environment in postpartum cows, although the exact strains and mechanisms remain unclear (Genís et al., 2017).

This study addresses these gaps by isolating and characterizing vaginal *Lactobacillus strains* from healthy Mongolian mares, with a focus on *Lactobacillus salivarius*—a species hypothesized to thrive in acidic, nutrient-poor niches due to its metabolic versatility. Using a polyphasic approach integrating phenotypic, biochemical, and 16S rRNA gene sequencing, we identified a novel strain, *Lactobacillus salivarius* Y20, and evaluated its probiotic potential through *in vitro* assays of acid/bile tolerance, antimicrobial activity against equine pathogens, and antioxidant capacity. To elucidate the genetic basis of these traits, we performed whole-genome sequencing and comparative genomic analysis, comparing Y20 with publicly available *Lactobacillus salivarius* genomes from diverse hosts and ecological niches. Our findings not only advance understanding of equine vaginal microbiota resilience but also introduce Y20 as a candidate probiotic for mitigating RTIs in mares. By bridging microbiology, genomics, and veterinary medicine, this work underscores the translational potential of equine-derived probiotics in sustainable livestock management and One Health approaches.

## 2 Materials and methods

### 2.1 Headings ethics statement

This study was approved by the Ethics Committee of the Inner Mongolia Agricultural University (NND2022046), dated 2 March 2022. All experimental procedures adhered to the guidelines for the care and use of experimental animals.

### 2.2 Isolation and screening of vaginal *Lactobacilli*

#### 2.2.1 Sample collection

In this study, the sampling subjects were female Mongolian horses (aged 2–3 years old) from the Salaqi Horse Farm in Baotou City, Inner Mongolia. All horses were confirmed to be healthy and disease-free through veterinary examination. After cleaning the vulva of each mare, vaginal secretions were collected using vaginal swabs. The secretions were then placed into sterile anaerobic bags, transported in an icebox, and processed in a sterile laminar flow hood at the laboratory of Inner Mongolia Agricultural University.

#### 2.2.2 Isolation and purification

##### 2.2.2.1 Dilution and plating

Swab samples were suspended in 50 mL phosphate-buffered saline (PBS), vortexed, and serially diluted (10^−1^ to 10^−6^). Aliquots (200 μL) of 10^−4^, 10^−5^, and 10^−6^ dilutions were spread onto MRS agar plates containing bromocresol purple (0.04 g/L). The MRS Broth (Qingdao Hopebio, HB0384-1) and Bromocresol Purple Agar (Qingdao Hopebio, HB8616-2) used in this step were sourced from commercial suppliers. The plates were incubated anaerobically at 37°C for 24–36 h.

##### 2.2.2.2 Preservation

Yellow-pigmented colonies (indicative of lactic acid production) were streaked onto fresh MRS agar and subcultured in MRS broth (37°C, 18–24 h) before storage in 20% glycerol at −80°C.

#### 2.2.3 Phenotypic characterization

##### 2.2.3.1 Litmus milk test

Twenty microliters of activated *Lactobacilli* (OD_600_ = 1.0) was inoculated into litmus milk medium and incubated anaerobically at 37°C for 24–36 h. Pink coagulation indicated acid production and casein precipitation.

##### 2.2.3.2 Acid tolerance

*Lactobacilli* were grown in MRS broth adjusted to pH 2.0, 3.0, or 4.0. OD_600_ was measured at 0, 2, 4, 6, 8, 12, 16, 24, and 32 h using the Infinite 200 PRO Microplate Reader (Tecan).

##### 2.2.3.3 Growth curve

*Lactobacilli* were incubated in MRS broth, and OD_600_ was recorded at the same time intervals.

##### 2.2.3.4 Probiotic traits

(1) Antimicrobial activity: Assessed using the Oxford cup method against quality control strains. The quality control strains used in this study and their sources are as follows: *Salmonella enterica* (CMCC (B) 50071), *Escherichia coli* (ATCC 25922), *Staphylococcus aureus* (CMCC (B) 26003), *Gardnerella vaginalis* (ATCC 49145), *Streptococcus equi* subsp. *zooepidemicus* (ATCC 39920), *Streptococcus pyogenes* (CMCC (B) 32210), inhibition zones were measured after 24–36 h of incubation. (2) Hydrogen peroxide production: *Lactobacilli* were streaked onto TMB-HRP-MRS agar and incubated anaerobically (36 h) followed by aerobic exposure (1 h). Blue-green coloration indicated H_2_O_2_ production. (3) Antioxidant capacity: Total superoxide dismutase (T-SOD), total antioxidant capacity (T-AOC), and DPPH radical scavenging were quantified using commercial kits (Nanjing Jiancheng Bioengineering Institute).

#### 2.2.4 Morphological and biochemical identification

##### 2.2.4.1 Gram staining

*Lactobacilli* were Gram-stained and examined under a light microscope (Nikon NI-U).

##### 2.2.4.2 Biolog GEN III MicroPlate

Pure cultures were suspended in IF-A inoculating fluid (Biolog) to 95% transmittance and incubated at 37°C for 24 h. The Biolog GEN III MicroPlate (Biolog Inc.) was used for this analysis. Metabolic profiles were analyzed using Biolog MicroLog software (Cao et al., 2025).

### 2.3 Genomic DNA extraction and preparation

The genomic investigation of *Lactobacillus salivarius* Y20 began with the isolation and cultivation of the strain. Single colonies were obtained by streak-inoculating *Lactobacillus salivarius* Y20 onto LB agar plates, followed by incubation at 37°C for 12–18 h. These colonies were then transferred to LB liquid medium and cultured overnight at 37°C with shaking at 110 rpm. Genomic DNA extraction was performed using the Biomed Gene Genomic DNA Extraction Kit (Model DL111-01), adhering to the manufacturer’s protocol to ensure high-quality DNA for downstream sequencing applications (Liang, 2021).

### 2.4 Whole-genome sequencing and data quality control

A hybrid sequencing approach was employed to comprehensively characterize the *Lactobacillus salivarius* Y20 genome, utilizing both second-generation (Illumina MiSeq) and third-generation (PacBio) sequencing technologies. The Whole Genome Shotgun (WGS) strategy was used for library construction, ensuring broad genomic coverage.

The Illumina sequencing data underwent rigorous quality control using Fast QC software. Parameters evaluated included GC content distribution, base quality distribution, sequence base quality, and base content distribution to ensure data integrity and suitability for downstream assembly and analysis (Liang, 2021).

### 2.5 Genome assembly and refinement

Given the extended read lengths provided by third-generation sequencing, the Falcon assembler was selected for *de novo* assembly of the PacBio reads. This approach simplified the assembly process and enhanced genome continuity.

#### 2.5.1 Assembly assessment and refinement

The resulting assembled genome was statistically analyzed, and second-generation sequencing data were used to refine the assembly, correcting potential errors. Assembly quality was assessed by aligning the original reads to the assembled genome, with coverage depth and GC distribution serving as key metrics.

#### 2.5.2 Genome visualization

A circular genome map was generated to provide a holistic and intuitive overview of the genomic architecture, facilitating the identification of key genomic features and structural elements (Liang, 2021).

### 2.6 Functional annotation and bacteriocin gene analysis

Functional annotation of the coding proteins within the *Lactobacillus salivarius* Y20 genome was conducted using a curated set of databases, including Nr, SwissProt, KEGG, GO, COG, Pfam Scan, and CAZy.

#### 2.6.1 Multi-database functional annotation

This approach enabled a comprehensive understanding of gene functions and metabolic pathways encoded within the genome, providing insights into the strain’s physiological and biochemical capabilities.

#### 2.6.2 Bacteriocin gene prediction and validation

Bacteriocin gene clusters were predicted using AntiSMASH, and the gene sequences encoding bacteriocins were further validated using BAGEL4, offering insights into the strain’s antimicrobial potential and ecological role (Blin et al., 2019).

### 2.7 Comparative genomics and evolutionary relationships

To contextualize the genomic features of *Lactobacillus sali*var*ius* Y20 within its broader taxonomic framework, comparative genomics analyses were undertaken.

#### 2.7.1 Reference genome selection

Existing genome assembly and annotation reports were sourced from the NCBI database^1^ to serve as reference genomes for comparative analysis.

#### 2.7.2 SNP and InDel detection

Single nucleotide polymorphisms (SNPs) were detected using the MUMmer alignment software, with statistical analysis conducted to elucidate their positional relationships with genes and mutation outcomes. Small-scale insertions and deletions (InDels, <50 bp) were identified using MUMme software, providing a detailed account of genomic variability.

#### 2.7.3 Synteny and structural variation analysis

Synteny analysis was performed to compare the evolutionary distances between *Lactobacillus salivarius* Y20 and reference genomes, with MUMmer used to identify large-scale syntenic relationships and SyRI for fine-scale alignment to detect chromosomal rearrangements (translocations, inversions, and combined translocations with inversions). Structural variations (SVs), encompassing insertions, deletions, inversions, and translocations of genomic segments >50 bp, were detected using SyRI software, offering insights into large-scale genomic rearrangements (Kurtz et al., 2004).

### 2.8 Population evolutionary analysis

To further elucidate the evolutionary relationships of *Lactobacillus salivarius* Y20, population evolutionary analyses were conducted.

#### 2.8.1 Gene family analysis and coreness

Gene family analysis involved aligning protein (or nucleotide) sequences from all analyzed species using diamond, followed by similarity clustering with OrthoMCL to generate lists of homologous genes (clusters). The species distribution within each cluster was statistically analyzed to infer evolutionary patterns. Gene coreness analysis focused on identifying species-specific genes in *Lactobacillus salivarius* Y20 and closely related strains, based on gene family clustering results, highlighting unique genomic features.

#### 2.8.2 Phylogenetic tree construction

A maximum-likelihood phylogenetic tree was constructed for the sequenced and reference strains using single-copy gene families identified in their whole genomes. The tree, built using iqtree, provided a robust framework for inferring evolutionary relationships among the strains.

#### 2.8.3 Average nucleotide identity analysis

The average nucleotide identity (ANI) between the sequenced and reference strains was calculated using pyani, offering a quantitative measure of genomic relatedness and further supporting the phylogenetic inferences.

### 2.9 Statistical analysis

The statistical analysis of the data was conducted using MEGA version 6.06 and SPSS version 23.0. For comparing means across multiple groups, a one-way analysis of variance (ANOVA) was employed, followed by Tukey’s *post hoc* test to identify specific pairwise differences. Visual representations of the data were generated using GraphPad Prism version 8 (Zhang et al., 2025).

## 3 Results

### 3.1 Isolation and screening of vaginal *Lactobacilli* from Mongolian mares

**Figure 1:**
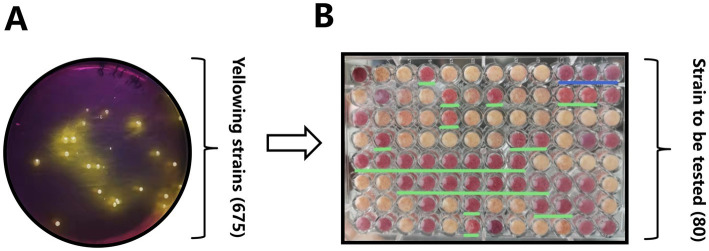
Initial screening pipeline for vaginal *Lactobacillus* strains from Mongolian mares. **(A)** An example of a bromocresol violet screening plate, where colonies producing lactic acid turn the medium yellow. **(B)** Results of the litmus milk color test, highlighting colonies with proteolytic activity and acidification potential.

**Figure 2:**
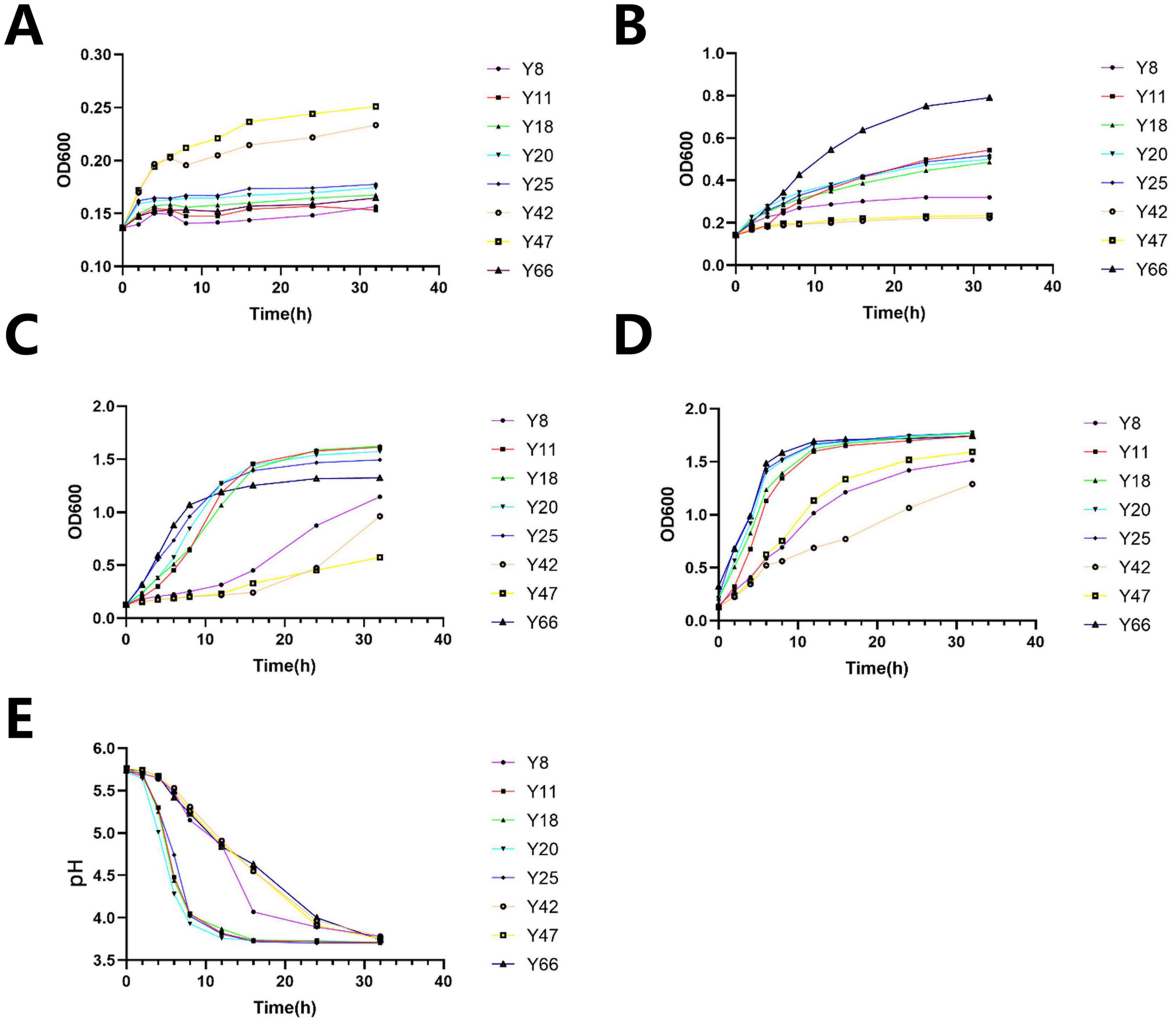
Results of acid resistance tests, growth assays, and acid production experiments. **(A)** Growth manifestation of bacterial strains under pH 2 condition: temporal variation of OD_600_ values. **(B)** Growth manifestation of bacterial strains under pH 2 condition: temporal variation of OD_600_ values. **(C)** Growth manifestation of bacterial strains under pH 4 condition: temporal variation of OD_600_ values. **(D)** Growth manifestation of bacterial strains: comprehensive temporal OD_600_ profiles. **(E)** Acid production manifestation of bacterial strains: temporal variation of pH values.

**Figure 3:**
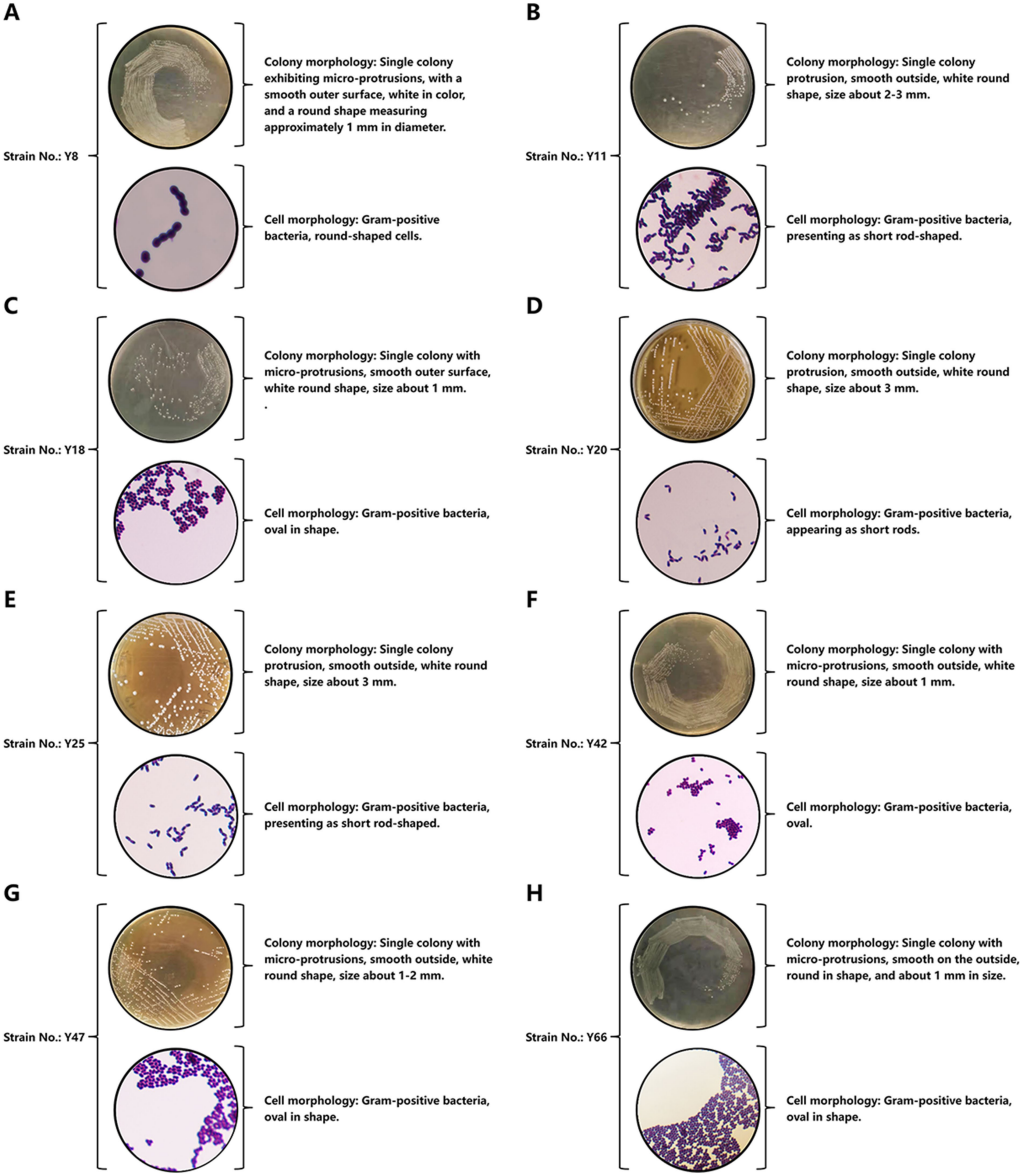
Morphological identification results. **(A)** Colony morphology and cell morphology of bacterial strain Y8. **(B)** Colony morphology and cell morphology of bacterial strain Y11. **(C)** Colony morphology and cell morphology of bacterial strain Y18. **(D)** Colony morphology and cell morphology of bacterial strain Y20. **(E)** Colony morphology and cell morphology of bacterial strain Y25. **(F)** Colony morphology and cell morphology of bacterial strain Y42. **(G)** Colony morphology and cell morphology of bacterial strain Y47. **(H)** Colony morphology and cell morphology of bacterial strain Y66.

Through a systematic approach, this part of the results describes the isolation, screening, and identification of an optimal vaginal *Lactobacillus* strain (Y20) from Mongolian mares. This was achieved by evaluating acid production, resistance, growth kinetics, and morphological traits (Figures 1–3).

Initially, 675 acid-producing colonies were isolated through dilution-plating on a bromocresol purple-containing medium. Color changes from purple to yellow (Figure 1A) served as an indicator of successful lactic acid production. Among these colonies, 80 strains (Y1-Y80) were selected using a litmus milk color test. In this test, a pink curd-like morphology (highlighted in the green-lined regions of Figure 1B) was found to correlate with proteolytic activity and acidification potential.

Subsequent assays were conducted to assess the strains’ growth under acidic stress (pH 2–4). The results showed that Y42 and Y47 exhibited superior growth at pH 2, with OD_600_ values of 0.25–0.23 (Figure 2A). Meanwhile, Y66 demonstrated excellent growth at pH 3, reaching an OD_600_ of 0.8 (Figure 2B). At pH 4, Y11, Y18, and Y20 achieved comparable growth, with OD_600_ values around 1.7 (Figure 2C), while Y25 had a slightly lower OD_600_ of 1.5. In the blank medium, Y20, Y25, and Y66 entered the logarithmic phase by 6 h, with an OD_600_ of 1.8 at 32 h (Figure 2D), outperforming the other strains. Notably, Y20 acidified the medium most rapidly, reaching a pH of 3.7 within 12 h, with a ΔpH of 2.0 (Figure 2E). It also maintained lower pH values (≤4.1 at 16 h) compared to all its competitors.

Morphological analysis (Figure 3) classified Y8, Y18, Y42, Y47, and Y66 as round or oval in shape (Figures 3A,C,F–H), while Y11, Y20, and Y25 were short-rod-shaped (Figures 3B,D,E). This morphological classification was consistent with their growth and acidification profiles. Given Y20’s exceptional acid production capabilities, its resilience to pH values ranging from 2 to 4, and its rapid growth, it was prioritized for advanced investigation into equine vaginal microbiome dynamics and probiotic applications. This highlights its critical role in maintaining vaginal health and contributing to the overall well-being of the host.

### 3.2 Identification results of vaginal *Lactobacilli* in Mongolian mares

**Figure 4:**
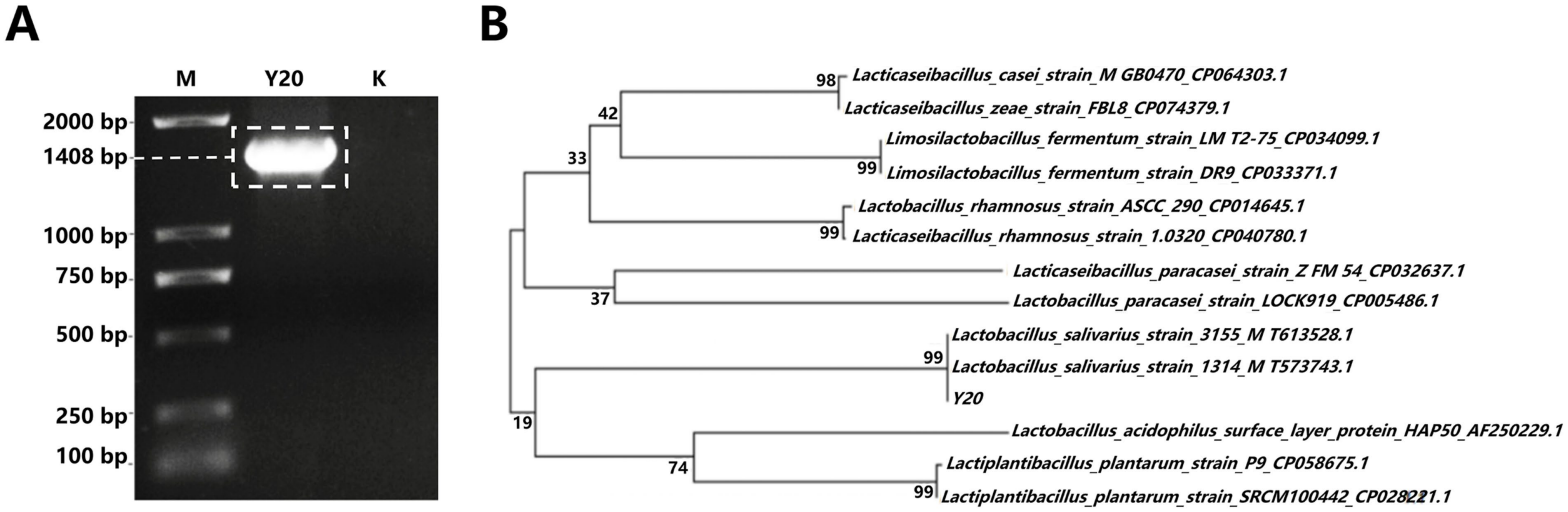
Electrophoresis diagram and phylogenetic tree. **(A)** Gel electrophoresis diagram of 16S rRNA amplification products, where “M” denotes the DNA Marker (DL2000) and “K” indicates the negative control. **(B)** Phylogenetic tree constructed based on 16S rRNA sequence homology, showing the evolutionary relationship of the isolated strain with other Lactobacillus species.

The identification results of the vaginal *Lactobacillus* strain Y20 from Mongolian mares are as follows (Figure 4 and Tables 1–3).

In the physiological and biochemical tests, strain Y20 was capable of fermenting salicin, sorbitol, sucrose, raffinose, inulin, and lactose, and it might also ferment cellobiose and mannitol. It did not produce indole, gelatinase, or H_2_S, nor could it hydrolyze starch (Table 1). Based on these results and in conjunction with the data from Bergey’s Manual of Determinative Bacteriology (9th edition), strain Y20 was preliminarily identified as belonging to the genus *Lactobacillus*.

In the Biolog microwell plate bacterial identification, after the Biolog microwell plate reaction, there were 38 positive reactions, 47 negative reactions, and 11 intermediate-value reactions (Supplementary Figure 1). Positive reactions were observed for substrates such as raffinose, maltose, trehalose, and sucrose, while negative reactions were observed for substrates like arabitol and inositol. Intermediate-value reactions were noted for substrates such as cellobiose and turanose. After integrating all the reactions and using the Biolog bacterial identification system, the highest similarity index (SIM) was 0.682 (>0.5), indicating reliable data. The strain with the highest SIM value was identified as *Lactobacillus salivarius*. Specifically, the top four strains with the highest SIM values were *Lactobacillus salivarius*, *Lactobacillus salivarius* ss *araffinosus*, *Leuconostoc mesenteroides* ss *dextranicum*, and *Lactobacillus brevis* (Table 2).

**Table 1:** Biochemical test result table of Y20.

Physiological and biochemical indicator	Y20
Esculin	−
Cellobiose	−/+
Maltose	+
Mannitol	−/+
Salicin	+
Sorbitol	+
Sucrose	+
Raffinose	+
Inulin	+
Lactose	+
1% Sodium hippurate	−
Indole	−
Starch hydrolysis	−
Catalase	−
Gelatin	−
H₂S	−
Motility	−

**Table 2:** Biolog microwell plate identification results.

Comparison number	PROB value	SIM value	DIST value	Biological type	Species
1	0.967	0.682	4.523	GP-Rod	*Lactobacillus salivarius*
2	0.018	0.139	5.566	GP-Rod	*Lactobacillus salivarius* ss *araffinosus*
3	0.008	0.094	6.207	GP-Coccus	*Leuconostoc mesenteroides* ss *dextranicum*
4	0.007	0.085	6.292	GP-Rod	*Lactobacillus brevis*

For the 16S rRNA gene identification, during the 16S rRNA gene PCR electrophoresis detection, analysis of the amplified products via 1.2% agarose gel electrophoresis revealed a distinct and singular target band of approximately 1,408 bp for strain Y20, aligning with the anticipated size (Figure 4A); subsequently, in the 16S rRNA gene PCR product sequencing and homology comparison, the PCR products were dispatched to BGI Genomics for sequencing, and BLAST software was employed to assess the homology of the 16S rRNA gene sequence of strain Y20 (Table 3), with the construction of a phylogenetic tree using MEGA6.06 indicating that strain Y20 exhibited the closest phylogenetic relationship to *Lactobacillus salivarius*, sharing a homology exceeding 99% (Figure 4B).

**Table 3:** 16S rRNA sequence homology alignment.

Serial number	English name	Coverage	Similarity	Gene amplification fragment size
Y20	*Lactobacillus salivarius*	100%	100%	1,408 bp

### 3.3 Analysis of biological characteristics of *Lactobacillus salivarius* Y20 isolated from the vagina of Mongolian mares

**Figure 5:**
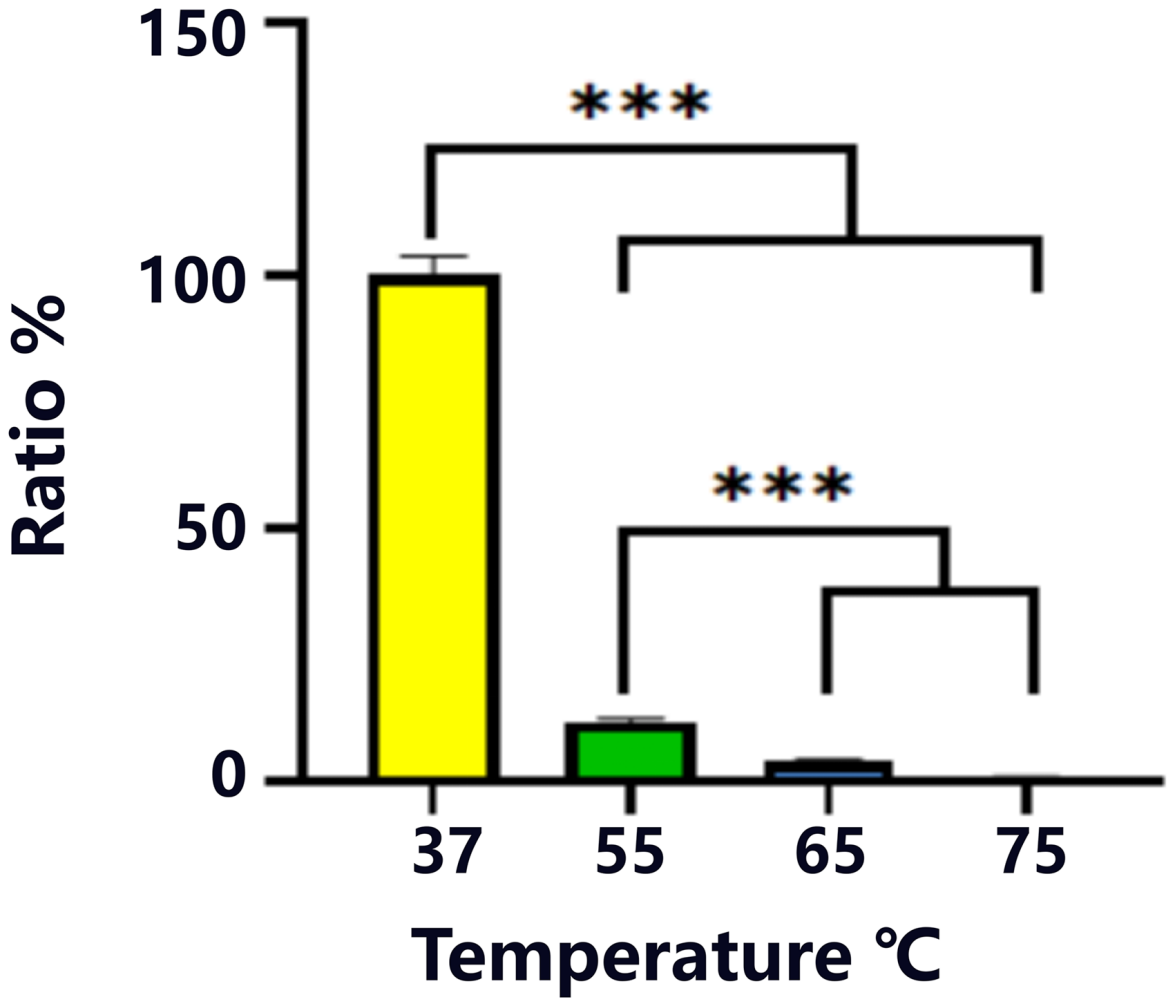
Heat resistance test for strain Y20. ^***^Indicates statistical significance at *p* < 0.001.

**Figure 6:**
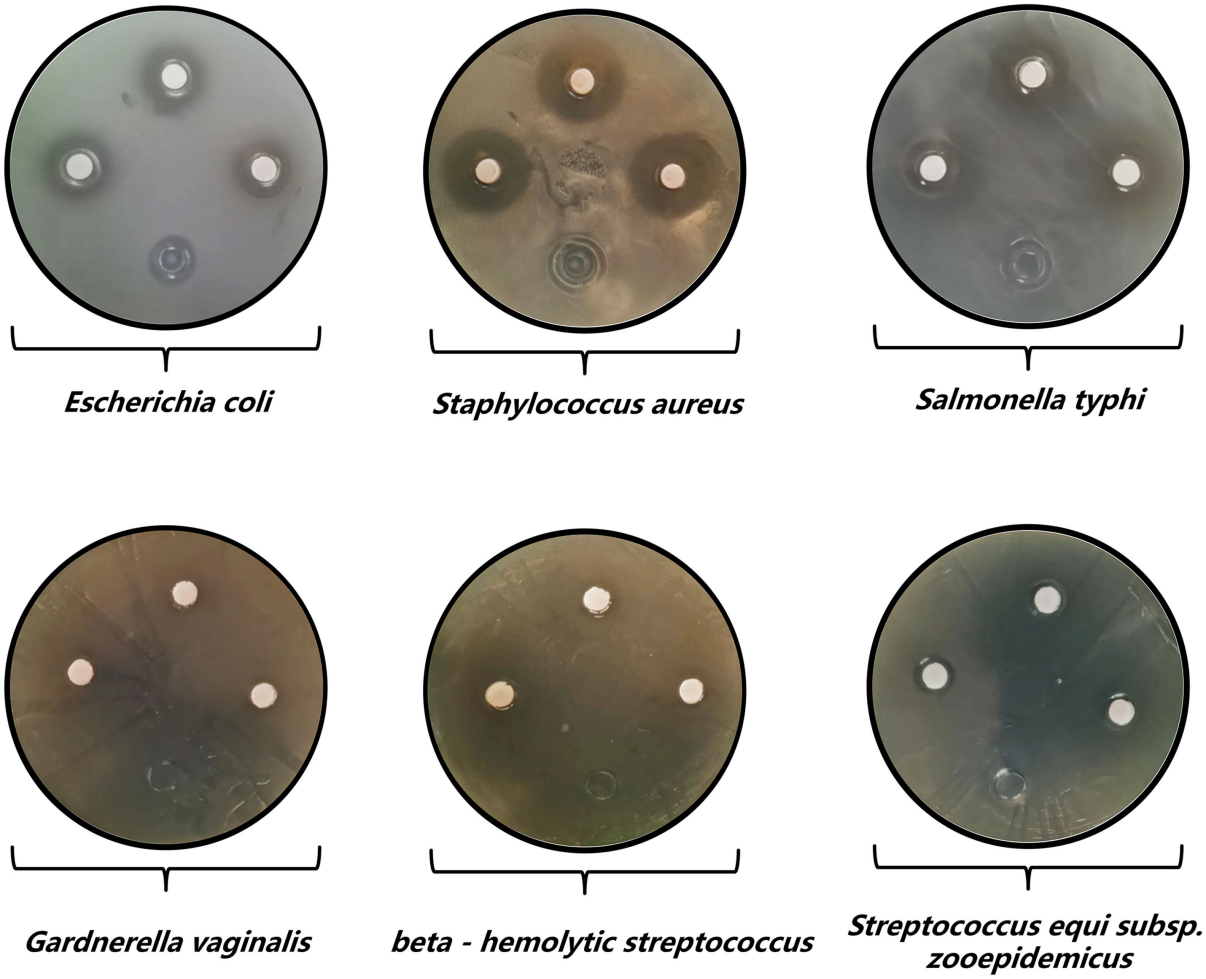
Antibacterial activity results of strain Y20.

**Figure 7:**
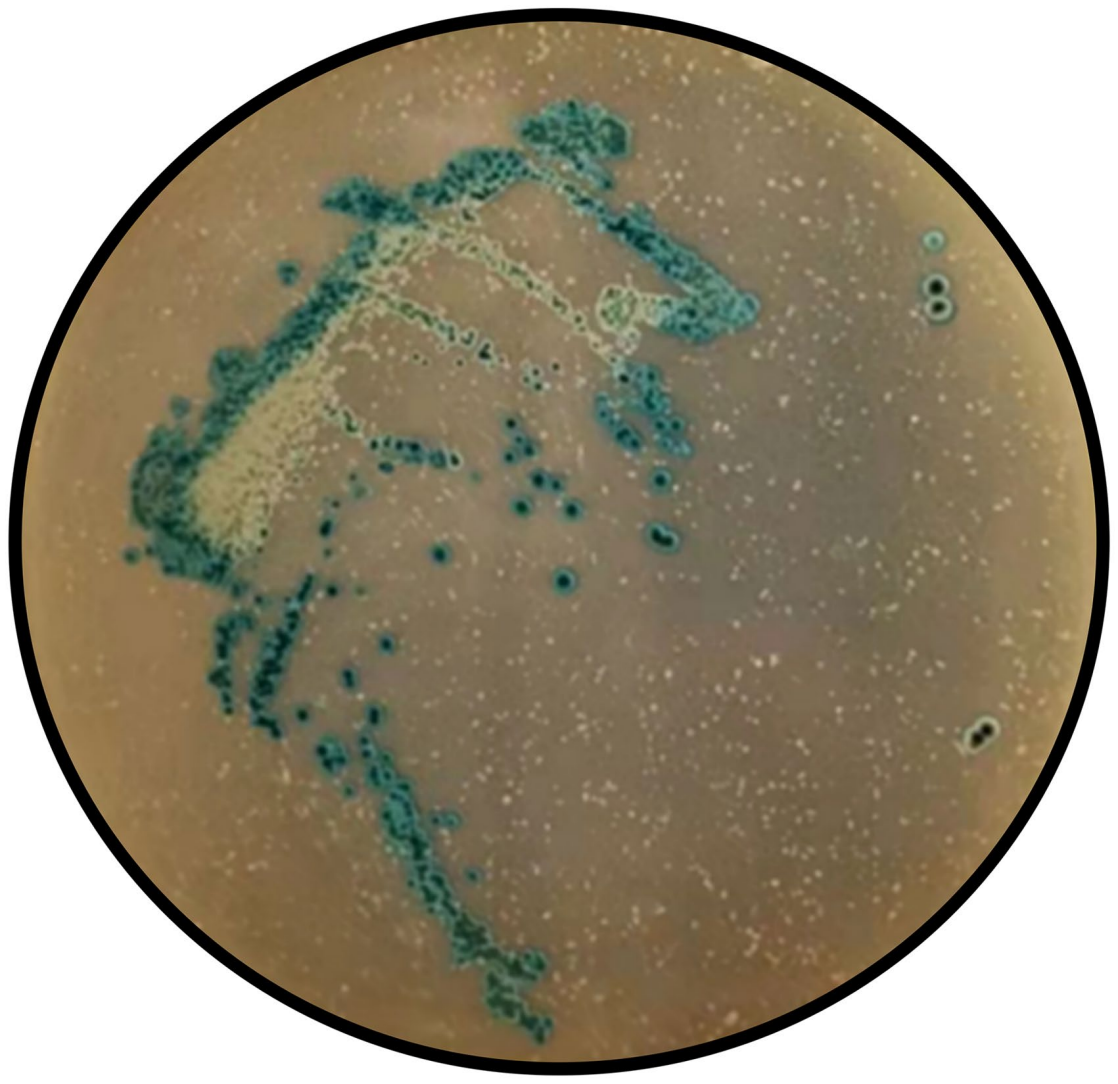
Hydrogen peroxide production test results of Y20.

**Figure 8:**
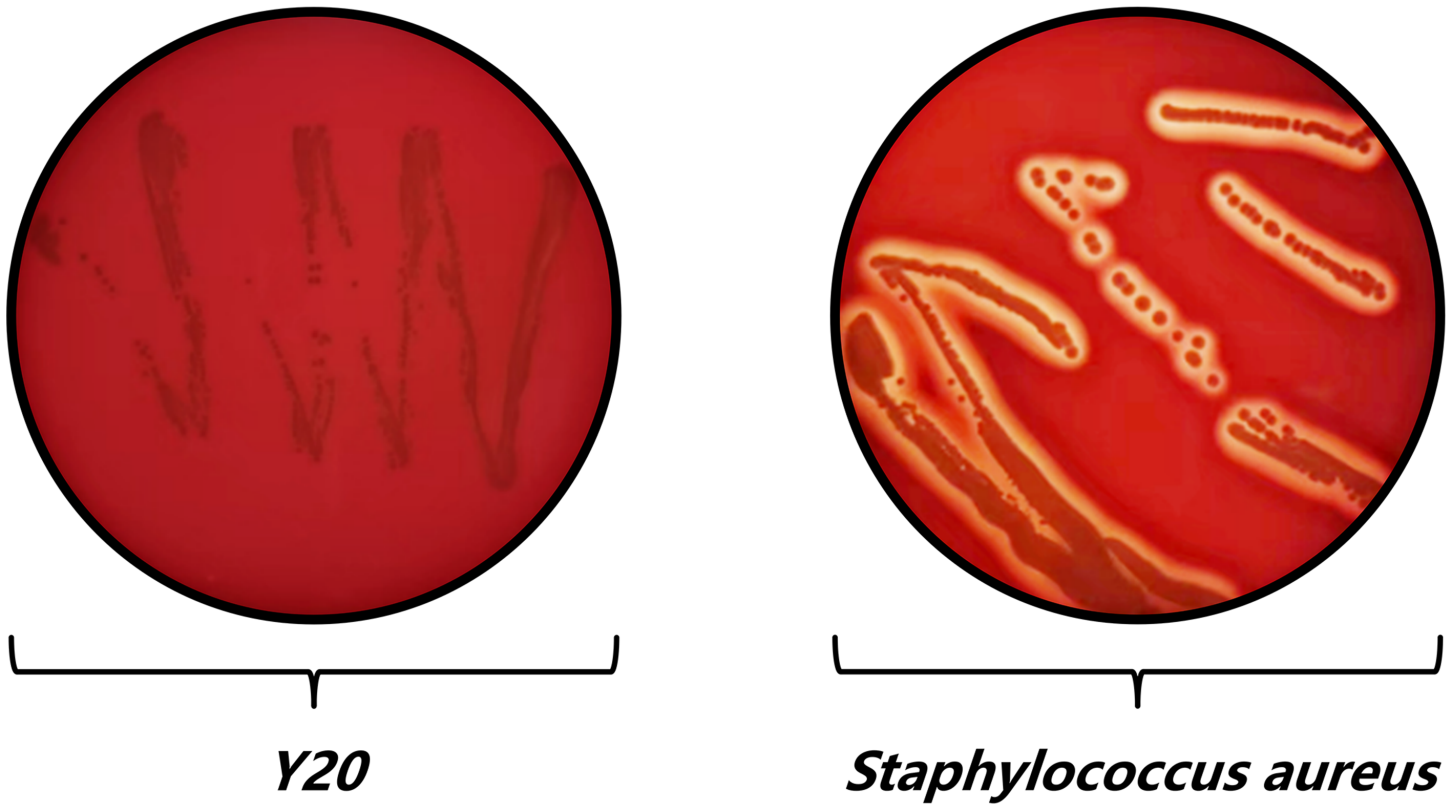
Hemolysis test results of Y20.

**Figure 9:**
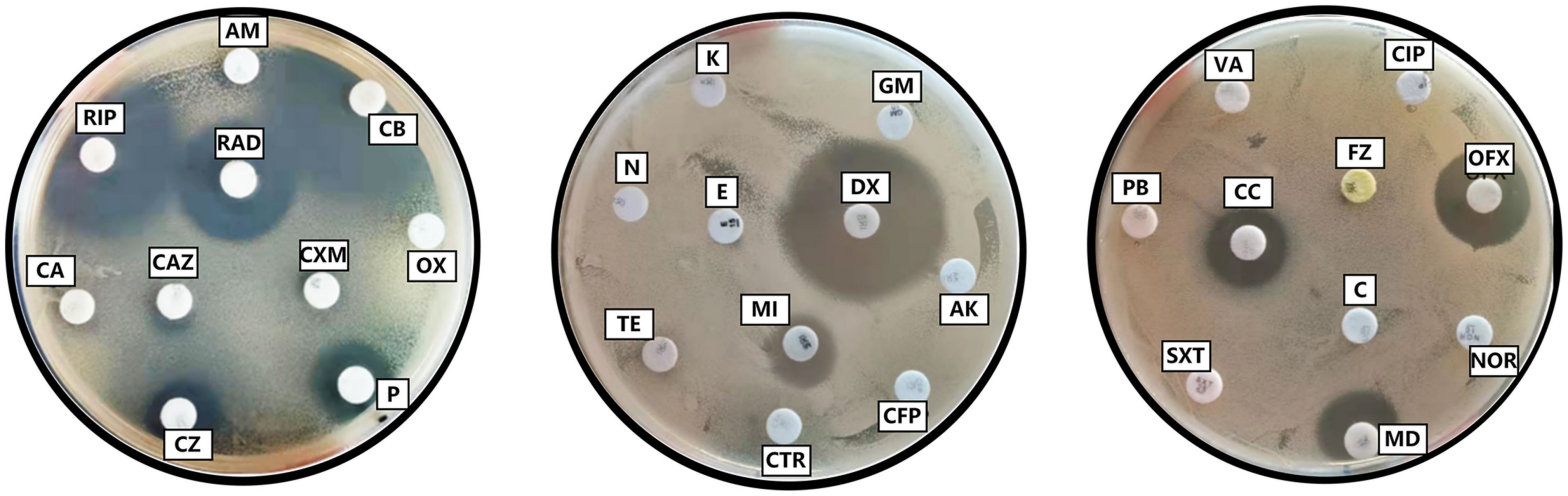
Antibiotic susceptibility test results of Y20. Penicillin (P), oxacillin (OX), carbenicillin (CB), ampicillin (AM), piperacillin (PIP), cefalexin (CA), cefazolin (CZ), cefradine (RAD), cefuroxime (CXM), ceftazidime (CAZ), ceftriaxone (CTR), cefoperazone (CFP), amikacin (AK), gentamicin (GM), kanamycin (K), neomycin (N), tetracycline (TE), doxycycline (DX), minocycline (MI), erythromycin (E), midecamycin (MD), norfloxacin (NOR), ofloxacin (OFX), ciprofloxacin (CIP), vancomycin (VA), polymyxin B (PB), sulfamethoxazole-trimethoprim (SXT), furazolidone (FZ), chloramphenicol (C), clindamycin (CC).

A meticulous and multifaceted evaluation of the biological attributes of *Lactobacillus salivarius* Y20, isolated from the vaginal microenvironment of Mongolian mares, was undertaken utilizing a series of rigorously designed and executed assays (Figures 5–9 and Tables 4, 5).

During the heat-resistance assessment, the heat-survival ratio of strain Y20 was precisely determined, with 37°C serving as the reference survival rate (Figure 5). A striking and statistically robust decline (*p* < 0.001) in the survival rate was observed as the temperature was elevated to 55°C, 65°C, and 75°C. At the extreme temperature of 75°C, the survival rate of Y20 experienced a precipitous drop to less than 1%, clearly indicating its relatively limited thermotolerance.

Shifting focus to the antibacterial testing, strain Y20 demonstrated a broad-spectrum antimicrobial activity against six well-established and thoroughly characterized quality-control strains (Table 4 and Figure 6). Among these, the most pronounced inhibitory effect was exerted against *Staphylococcus aureus*, as evidenced by an exceptionally large inhibition zone diameter of 20.42 mm. A hierarchical order of inhibitory efficacy was established, with *Escherichia coli* exhibiting the second-highest level of inhibition, succeeded by *Salmonella typhi*, *Streptococcus equi* subsp. *zooepidemicus*, *Gardnerella vaginalis*, and finally *Streptococcus hemolyticus* group B, which displayed the least inhibitory response among the tested strains.

**Table 4:** Antibacterial activity test results.

Bacterial strain	Inhibition zone diameter (mm) ± standard deviation
*Escherichia coli*	15.1600 ± 0.8795
*Staphylococcus aureus*	20.4233 ± 0.1297
*Salmonella enterica* serovar Typhi	14.0333 ± 0.4288
*Gardnerella vaginalis*	11.0066 ± 0.0903
*Streptococcus pyogenes* (Group A β-hemolytic)	10.4100 ± 0.0294
*Streptococcus equi* subsp. *zooepidemicus*	12.3333 ± 0.1271

In the hydrogen peroxide production assay, all colonies of Y20 exhibited a characteristic blue reaction (Figure 7), unequivocally indicating a positive result and underscoring its capacity to synthesize hydrogen peroxide, a crucial antimicrobial metabolite with significant implications for microbial competition and host defense. The antioxidant capacity analysis revealed that strain Y20 possesses remarkable antioxidant properties. It achieved a DPPH radical-scavenging rate of 73.77%, a total antioxidant capacity (T-AOC) of 67.49 U/mL, and a total superoxide dismutase activity (T-SOD) of 59.66 U/mL. These findings suggest its potential to effectively counteract oxidative stress within the vaginal environment, which is a critical factor in maintaining vaginal health and preventing oxidative-damage-related pathologies.

The hemolysis test provided compelling evidence of the safety profile of *Lactobacillus salivarius* Y20. In contrast to *Staphylococcus aureus*, which produced a distinct and well-defined hemolysis ring, strain Y20 did not generate any such ring (Figure 8). This observation unequivocally indicates its non-hemolytic nature, thereby offering a high level of assurance regarding its safety for potential therapeutic or probiotic applications.

In the drug-sensitivity analysis, a panel of 10 antibiotics, including penicillin, carbenicillin, piperacillin, cefazolin, cefradine, doxycycline, minocycline, midecamycin, ofloxacin, and clindamycin, exhibited inhibitory effects on strain Y20. The strain demonstrated a high degree of susceptibility to carbenicillin, piperacillin, cefradine, and doxycycline, while displaying moderate susceptibility to midecamycin, ofloxacin, and clindamycin. Conversely, it exhibited resistance to penicillin, cefazolin, and minocycline, and showed no susceptibility to the remaining 20 antibiotics included in the test panel (Table 5 and Figure 9).

**Table 5:** Drug sensitivity test results of Y20.

Antibiotic disk	Inhibition zone diameter (cm)/interpretation
Penicillin (P)	1.33/R
Oxacillin (OX)	-/R
Carbenicillin (CB)	3.24/S
Ampicillin (AM)	-/R
Piperacillin (PIP)	2.30/S
Cefalexin (CA)	-/R
Cefazolin (CZ)	1.26/R
Cefradine (RAD)	2.07/S
Cefuroxime (CXM)	-/R
Ceftazidime (CAZ)	-/R
Ceftriaxone (CTR)	-/R
Cefoperazone (CFP)	-/R
Amikacin (AK)	-/R
Gentamicin (GM)	-/R
Kanamycin (K)	-/R
Neomycin (N)	-/R
Tetracycline (TE)	-/R
Doxycycline (DX)	2.91/S
Minocycline (MI)	1.13/R
Erythromycin (E)	-/R
Midecamycin (MD)	1.45/I
Norfloxacin (NOR)	-/R
Ofloxacin (OFX)	1.69/I
Ciprofloxacin (CIP)	-/R
Vancomycin (VA)	-/R
Polymyxin B (PB)	-/R
Sulfamethoxazole-trimethoprim (SXT)	-/R
Furazolidone (FZ)	-/R
Chloramphenicol (C)	-/R
Clindamycin (CC)	1.42/I

S (Sensitive): Bacterial growth is fully inhibited within the susceptible breakpoint range. I (Intermediate): Bacterial growth is partially inhibited, requiring higher doses or alternative therapies. R (Resistant): Bacterial growth is not inhibited within the susceptible breakpoint range. “-”: No measurable inhibition zone observed (indicating resistance or methodological limitation).

With regard to the auto-aggregation and biofilm-formation capacity assessments, strain Y20 displayed an outstanding auto-aggregation rate (A%) of 86.8127%. In the biofilm-formation evaluation, the OD595 value of strain Y20 was measured at 1.2834, with an ODC of 0.4244. Given the criterion of 2 ODc < OD ≤ 4 ODc, this indicates a moderate (++) biofilm-formation ability. These attributes are of paramount significance for *Lactobacilli*, as they facilitate robust adhesion to vaginal epithelial cells, enhance their adaptability and survival within the complex vaginal ecosystem, and enable them to effectively compete with pathogenic bacteria, thereby playing a pivotal role in maintaining vaginal homeostasis and preventing infections.

### 3.4 Whole-genome analysis of *Lactobacillus salivarius* strain Y20

#### 3.4.1 Genome-wide visualization map

The genome of strain Y20 was assembled using Falcon, a long-read sequencing assembler, yielding a final genome size of 1,735,042 base pairs (bp) with a GC content of 33.01%. Detailed assembly metrics are provided in Supplementary Table 1. To assess GC bias, a GC-depth distribution plot was generated (Supplementary Figure 2), revealing a homogeneously clustered scatter pattern within a narrow range, indicating the absence of species contamination or GC skew in the Y20 genome. The 16S rRNA gene sequence of *Lactobacillus sali*var*ius* Y20 is presented in Supplementary Table 2.

**Figure 10:**
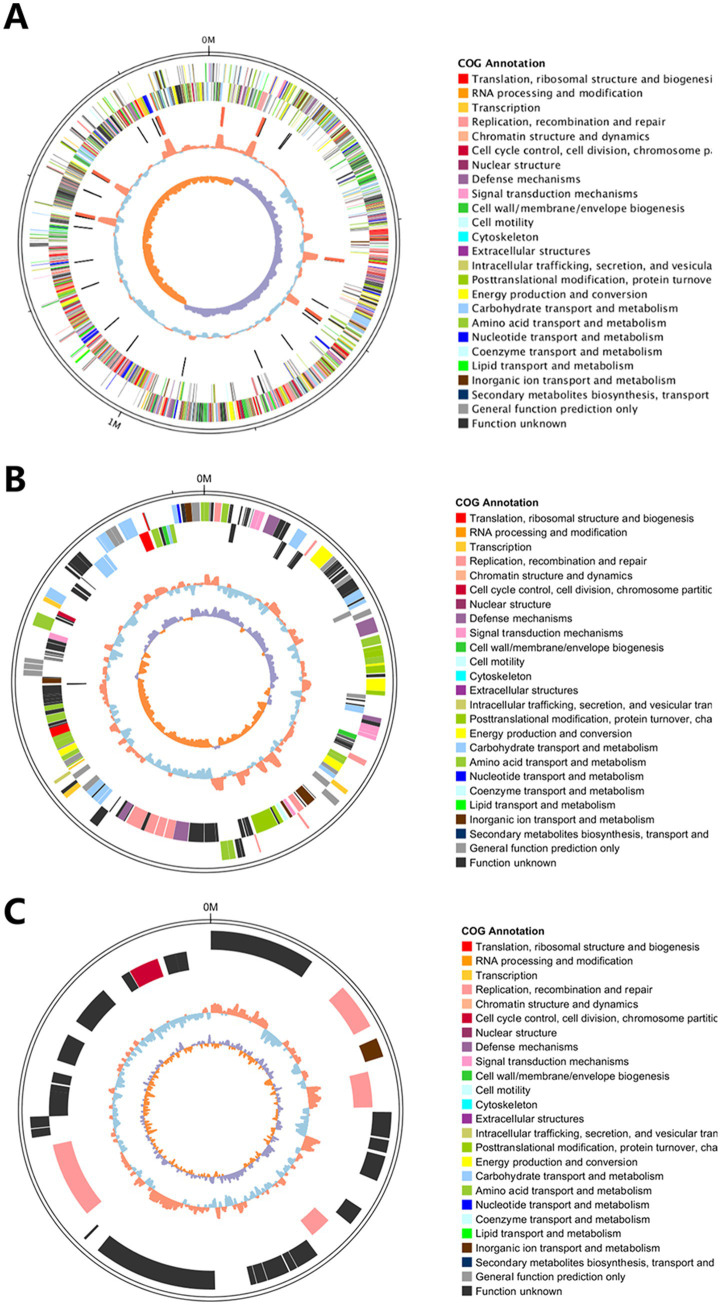
Circular genome and plasmid maps of Y20. **(A)** Genome circle map of Y20. From the outermost to the innermost layer: the outermost solid circle represents the genomic sequence position coordinates. Moving inward, the first circle illustrates the distribution of genes annotated by COG on the positive strand. The second circle inward depicts the distribution of genes annotated by COG on the negative strand. The third circle inward displays the distribution of ncRNAs, including tRNAs marked in black and rRNAs marked in red. The fourth circle inward represents the GC content, where orange indicates regions with GC content above the mean, and blue denotes regions with GC content below the mean. The fifth and innermost circle corresponds to the GC skew values, with specific color coding: orange for regions where the GC skew value is greater than 0 (GC skew >0), and purple for regions where the GC skew value is less than 0 (GC skew <0). **(B)** The outermost circle represents the positional coordinates of the genomic sequence. From the outermost to the innermost circles, the annotations are as follows: genes on the positive strand, genes on the negative strand, ncRNAs (where tRNAs are denoted in black and rRNAs in red), GC content (with red indicating values above the mean and blue indicating values below the mean), and GC skew (which reflects the relative abundance of G and C nucleotides and is used to identify the origin and terminus in circular chromosomes; it is calculated as GC skew = (G − C)/(G + C); purple denotes values greater than 0, while orange denotes values less than 0). **(C)** The outermost track displays the positional coordinates along the genomic sequence. Progressing inward from this outermost track, the subsequent circles depict, in order: genes located on the positive (forward) strand, genes on the negative (reverse) strand, non-coding RNAs (ncRNAs; with tRNAs represented in black and rRNAs in red), GC content (where red indicates regions with a GC proportion exceeding the mean, and blue denotes regions below the mean), and GC skew (a measure of the relative abundance of guanine (G) versus cytosine (C) nucleotides, which is instrumental in identifying the origin and terminus of replication in circular chromosomes. GC skew is calculated as (G − C)/(G + C); purple regions signify a positive GC skew value (>0), while orange regions indicate a negative skew value (<0)).

A circular genome map of Y20 was constructed, demonstrating that the assembly achieved a complete genome level (Figure 10A), with plasmids P1 and P2 visualized in Figures 10B,C, respectively. This integrated visualization—combining genomic architecture, plasmid content, and GC-depth profiling—provides a comprehensive and intuitive understanding of the structural and compositional features of the Y20 genome.

#### 3.4.2 Genomic composition analysis

**Figure 11:**
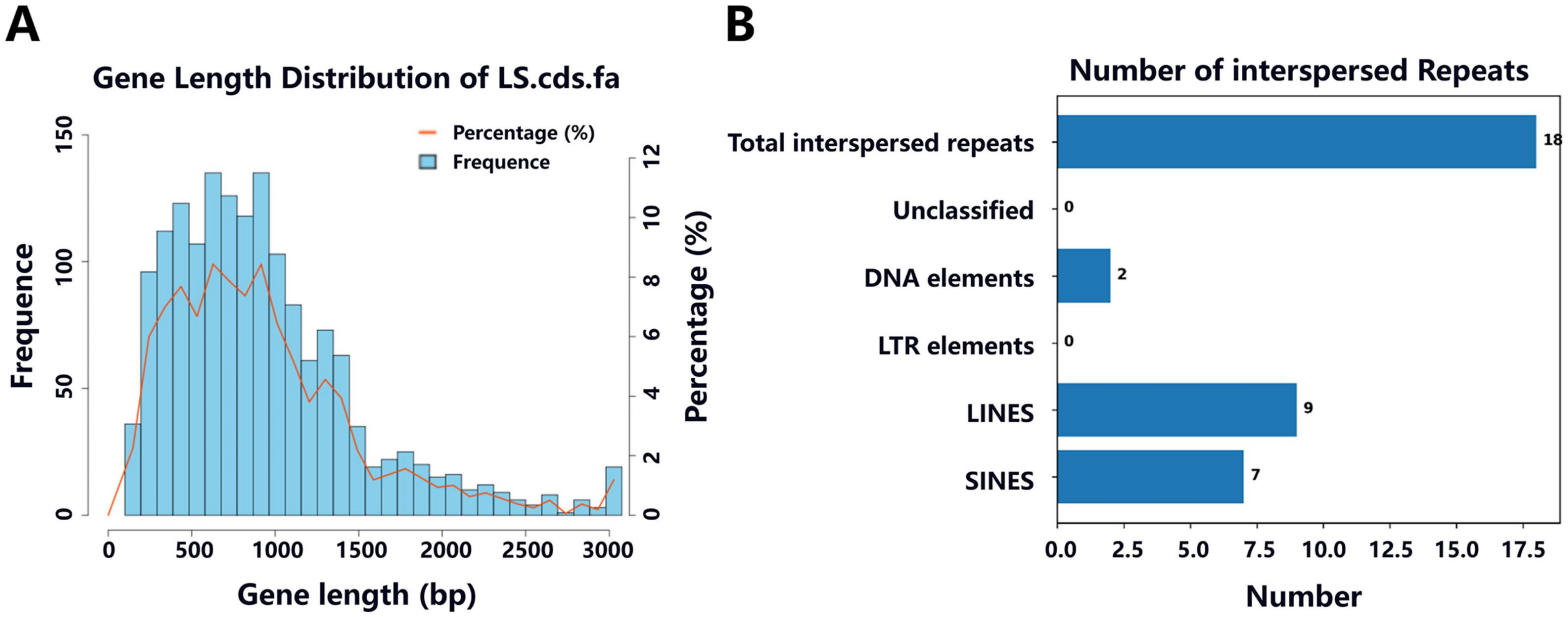
Length distribution of coding sequences and histogram analysis of interspersed repeats in the Y20 genome. **(A)** Length distribution of coding gene sequence. **(B)** Interspersed repeat histogram.

Predictive analyses of the Y20 genome were conducted using NCBI and Prokka, yielding the following results: in the coding gene prediction, a total of 1,602 genes were identified, with a cumulative gene length of 1,505,322 base pairs (bp). The longest gene spans 5,856 bp, while the shortest is 99 bp, with gene lengths predominantly clustering within the 200–1,400 bp range (Figure 11A). The overall genomic GC content was determined to be 33.27%. Regarding non-coding RNA prediction (Table 6), a total of 79 tRNAs and 22 rRNAs were identified. Predictive analysis of interspersed and tandem repeats (Figure 11B) revealed the presence of seven short interspersed nuclear elements (SINEs), each 471 bp in length, nine long interspersed nuclear elements (LINEs), each 818 bp in length, and two DNA transposons (DNA elements), each 87 bp in length. Transposon and CRISPR sequence predictions (Table 7) further indicated the discovery of six transposons and two CRISPR arrays. Predictions of genomic islands (GIs) and prophages (Table 8) demonstrated the existence of three GIs in the Y20 genome, with a cumulative length of 74,287 bp and an average length of 24,762.33 bp. Additionally, one prophage with a total length of 64,184 bp was detected.

**Table 6:** Statistics of non-coding RNA prediction results.

RNA type	Gene count
tRNA (transfer RNA)	79
rRNA (ribosomal RNA)	22
16S rRNA (16S ribosomal RNA)	7
5S rRNA (5S ribosomal RNA)	8
23S rRNA (23S ribosomal RNA)	7

**Table 7:** Transposon prediction and CRISPR sequence prediction results.

Genomic element type	Count
Transposons	6
CRISPR arrays	2

**Table 8:** Gene island and prophage prediction results.

Genomic element	Count	Total length (bp)	Average length (bp)
Genomic islands	3	74,287	24,762.33
Prophages	1	64,184	64,184.00

#### 3.4.3 Gene functional annotation

**Figure 12:**
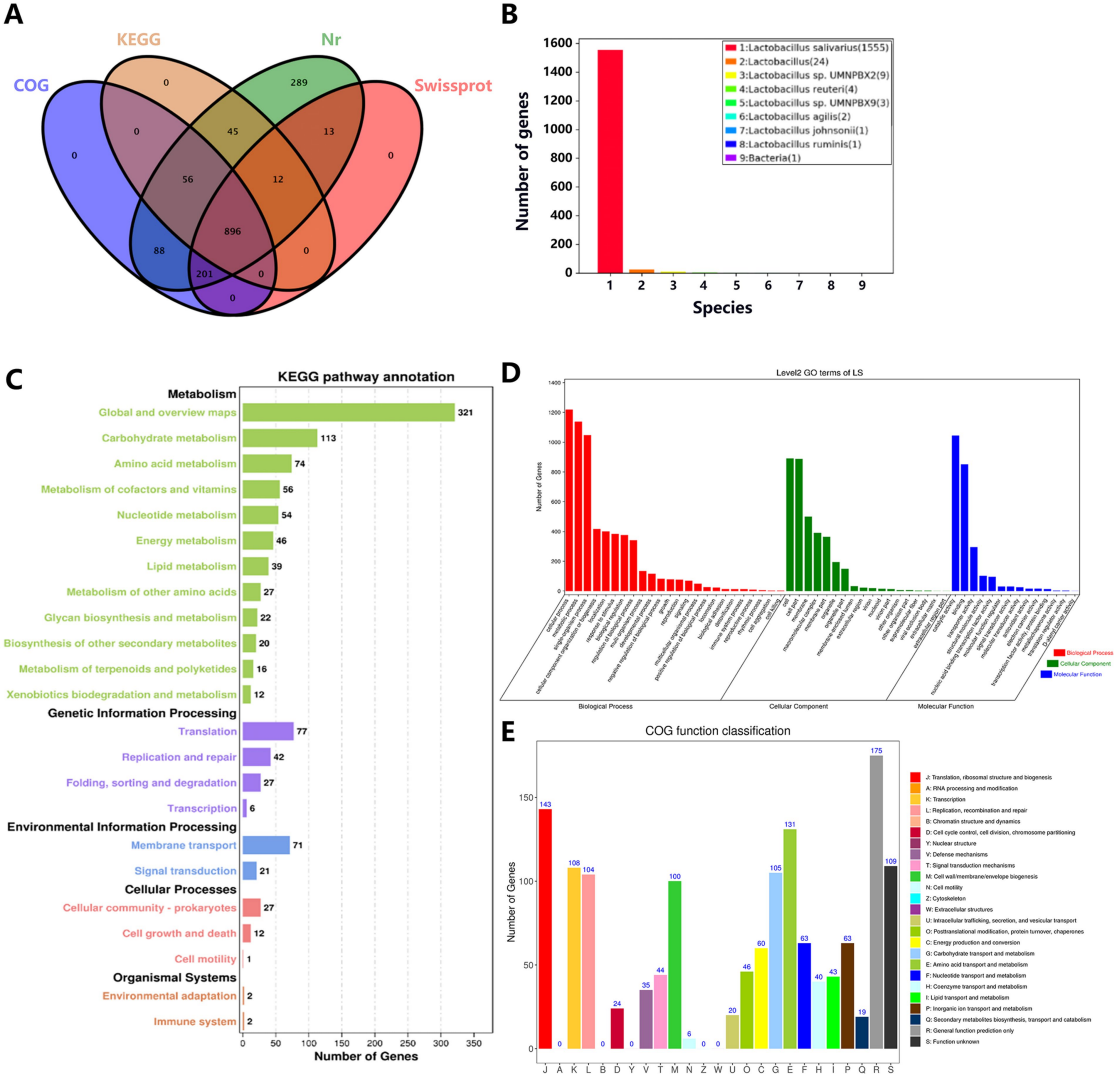
A comprehensive overview of functional annotation for Y20. **(A)** Four database annotation Venn diagrams. **(B)** Nr database annotated species statistics chart (Top 9 Species). **(C)** Statistics of KEGG pathway annotation results. **(D)** GO database function classification diagram. **(E)** COG annotation map.

The statistical data from the annotation functional databases reveal (Figure 12A) that among the genes of *Lactobacillus salivarius* Y20, 1,602 genes could be annotated, while the total number of unannotated genes was 2. In terms of annotation results from various databases, 1,600 genes were annotated in the Nr database, 1,122 genes in the Swissprot database, 1,241 genes in the KOG database, and 1,009 genes in the KEGG database. Notably, 896 genes were jointly annotated by all four databases. The Nr annotation results (Figure 12B) indicate that the highest number of genes (1,555) in *Lactobacillus salivarius* Y20 were homologous to those in *Lactobacillus salivarius*.

Based on the KEGG annotation information (Figure 12C), the functional classification of *Lactobacillus salivarius* Y20 comprises five hierarchical levels (KEGG A class). There are 800 genes associated with metabolism, 152 genes involved in genetic information processing, 92 genes related to environmental information processing, 40 genes linked to cellular processes, and four genes associated with organismal systems. Among them, immune-related pathways (such as the NOD-like receptor signaling pathway) are closely associated with the occurrence and development of multiple immune diseases. These pathways can also regulate the structure and function of the intestinal microbiota, thereby maintaining intestinal immune homeostasis. Additionally, the *trxA* gene, which encodes thioredoxin reductase, can modulate the redox state and protect DNA from oxidative damage.

Through GO database analysis (Figure 12D), a total of 12,076 gene annotation sequences were obtained, categorized into three major categories and 56 sub-entries. Specifically, 6,040 sequences were classified under biological process, 3,503 under cellular component, and 2,533 under molecular function. There are 17 genes associated with antioxidant activity, including thioredoxin-disulfide reductase and dihydrolipoamide dehydrogenase. Thioredoxin-disulfide reductase primarily participates in regulating the intracellular redox balance, while dihydrolipoamide dehydrogenase is a key enzyme that can convert organic matter into energy and provide power for vital activities.

The COG annotation results of *Lactobacillus salivarius* Y20 are classified into 26 functional categories (Figure 12E). Among them, there are 143 genes related to translation, ribosomal structure, and biogenesis; 104 genes involved in replication, recombination, and repair; 105 genes associated with carbohydrate transport and metabolism; and 131 genes related to amino acid transport and metabolism.

#### 3.4.4 Analysis of special functional elements

**Figure 13:**
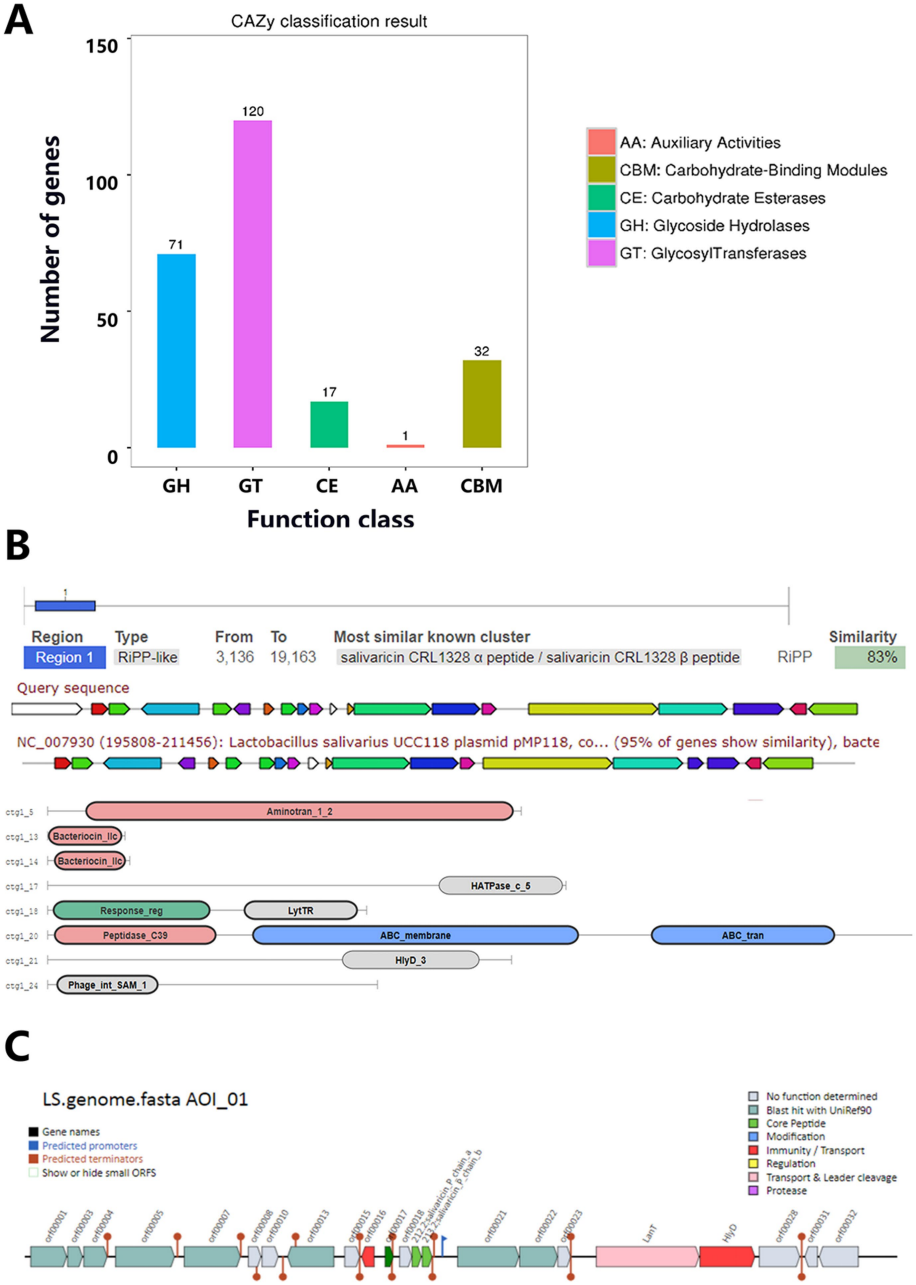
Special functional elements analysis for Y20. **(A)** CAZy classification chart. **(B)** Prediction of Y20 gene cluster. **(C)** Area of interest from Y20.

The CAZy annotation results of *Lactobacillus salivarius* Y20 revealed the presence of 241 enzymes belonging to five carbohydrate-enzyme-related categories, including 120 glycosyl transferases (GTs), 71 glycoside hydrolases (GHs), 32 carbohydrate-binding modules (CBMs), 17 carbohydrate esterases (CEs), and 1 auxiliary activity (AA) enzyme (Figure 13A). For the prediction of secretory proteins in Y20, 62 proteins with signal peptide structures, 35 proteins with transmembrane domains, and 27 proteins predicted to be secretory proteins were identified (Table 9). In the prediction of bacteriocin gene clusters in *Lactobacillus sali*var*ius* Y20, one bacteriocin gene cluster was found on the P1 plasmid. Sequence alignment showed that this bacteriocin gene cluster exhibited a high degree of similarity (83%) to the bacteriocin-encoding gene cluster in *Lactobacillus salivarius* UCC118 (Figure 13B). Through AntiSMASH analysis, three bacteriocins were identified within a single bacteriocin gene cluster on the plasmid genome. BAGEL analysis identified one bacteriocin hotspot region (Figure 13C). After database comparison, it was found that two of the bacteriocins showed high similarity to previously discovered bacteriocins, while one exhibited low similarity, suggesting that this bacteriocin might be a novel one (Table 10). Based on these findings, it can be inferred that *Lactobacillus salivarius* Y20 may produce a diverse range of bacteriocins.

**Table 9:** Secreted protein prediction statistics.

Sample name	Total protein	Signal peptide	Protein with transmembrane structure	Secreted protein
Y20	1,602	62	35	27

**Table 10:** Y20 bacteriocin gene situation table.

Name	Size	Nucleotide sequence	Amino acid sequence
Bacteriocin_IIc1	195 bp	ATGATGAAGGAATTTACAGTATTGACAGAATGTGAATTAGCAAAGGTTGATGGTGGGAAACGTGGACCTAACTGTGTAGGTAACTTCTTAGGTGGTCTATTTGCTGGAGCAGCTGCAGGAGTTCCACTTGGACCAGCTGGTATCGTAGGTGGGGCAAATCTAGGAATGGTAGGCGGAGCGCTTACTTGTTTATAA	MMKEFTVLTECELAKVDGGKRGPNCVGNFLGGLFAGAAAGVPLGPAGIVGGANLGMVGGALTCL
Bacteriocin_IIc2	207 bp	ATGAAAAATCTAGATAAAAGATTCACAATTATGACTGAAGATAACTTAGCATCAGTAAATGGTGGTAAAAATGGTTATGGTGGTAGCGGAAATCGCTGGGTTCACTGTGGAGCTGGCATCGTAGGTGGAGCTTTAATTGGAGCTATCGGTGGACCCTGGTCAGCCGTAGCGGGTGGAATTTCTGGTGGATTTGCAAGTTGCCATTAA	MKNLDKRFTIMTEDNLASVNGGKNGYGGSGNRWVHCGAGIVGGALIGAIGGPWSAVAGGISGGFASCH
Bacteriocin_IIc3	174 bp	ATGAATAATAATTTTGTACAAGTTGATAAGAAAGAATTGGCACATATAATTGGTGGAAGAAATTCTTATGATTATATAGATAGCGGACAGTTTGGTTACGATATAGGATGTACAATTGCTAATACTAAATTTTTCAAAAGATTAAGACACTCAAATCAAAATATTTGTAGTTAA	MNNNFVQVDKKELAHIIGGRNSYDYIDSGQFGYDIGCTIANTKFFKRLRHSNQNICS

#### 3.4.5 Comparative genomics analysis

After gaining insights into the gene composition and functional profile of *Lactobacillus salivarius* Y20, we aimed to further elucidate its taxonomic status and its relationships with closely-related species. To this end, complete genomes of *Lactobacillus salivarius* from diverse sources were retrieved from the NCBI database, and comprehensive analyses including whole-genome synteny, gene family, pangenome, and phylogenetic relationships were conducted. The reference strains selected are listed in Table 11.

**Table 11:** Genome summary of *Lactobacillus salivarius* from different sources.

Sequenced genome	Reference genome	Size (Mb)	Source
Y20	Lsa_IBB3154	2.17	Hen feces
	Lsa_JCM1046	2.32	Pig intestine
	Lsa_LPM01	2.03	Human milk
	Lsa_2D	1.98	Horse feces
	Lsa_S01	1.94	Fish intestine

**Figure 14:**
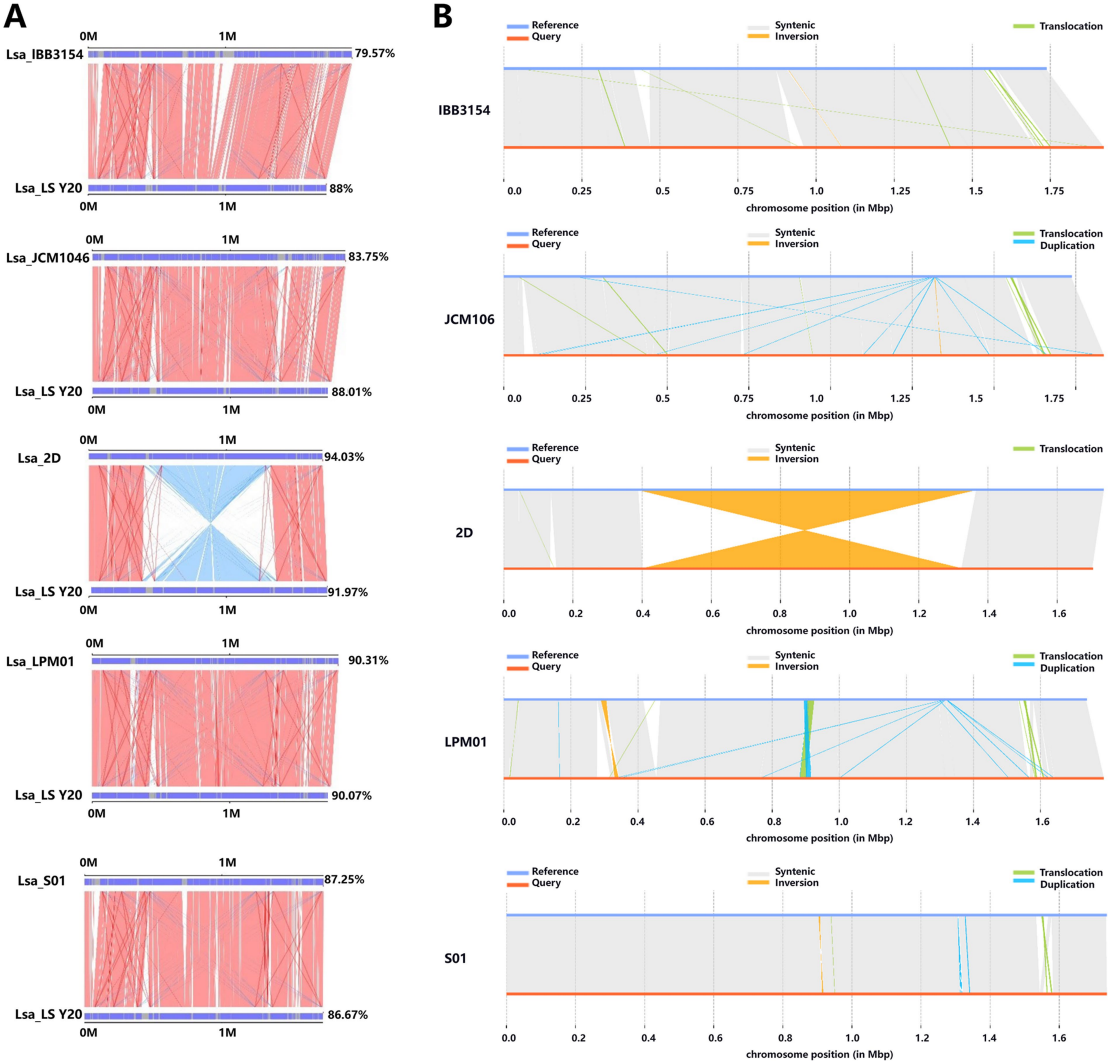
Comprehensive analysis of parallel collinearity and variation types. **(A)** Results of parallel collinearity. The upper axis represents the target species genome, and the lower axis represents the reference genome. Red lines: forward matches of corresponding regions. Blue lines: reverse matches of corresponding regions. **(B)** Display of variation types. Synthetic: collinear region. Translocation: translocation region. Inversion: inversion area. Insertion: insertion area. Deletion: deletion of region. Duplication: repeated area.

Parallel synteny plots were employed to display the syntenic relationships between the reference genomes and the target species (Y20) genome. By comparing the evolutionary distance disparities between pairwise species, we could analyze the phylogenetic relationships between the sample species (Y20) and the reference species. The synteny alignment results between *Lactobacillus salivarius* Y20 and the reference genomes indicated that IBB3154, LPM01, S01, and JCM1046 had fewer reverse-matching regions with Y20. In contrast, 2D exhibited more reverse-matching regions with Y20, with sizes ranging from 0.5–1.5 Mbp, suggesting that Y20 is most closely related to the equine-derived *Lactobacillus salivarius* 2D (Table 12 and Figure 14A).

**Table 12:** Statistics of coverage of collinearity comparison between reference strain and Y20.

Index	Strain ID				
	Lsa_IBB3154	Lsa_JCM1046	Lsa_LPM01	Lsa_2D	Lsa_S01
Total length of aligned target sequence region (bp)	1,526,770	1,526,969	1,578,263	1,595,818	1,503,801
Total length of target sequence (bp)	1,735,042	1,735,042	1,735,042	1,735,042	1,735,042
Percentage of aligned target sequence region in whole genome (%)	88	88.01	90.96	91.98	86.67
Total length of aligned query sequence region (bp)	1,528,844	1,537,891	1,615,186	1,599,332	1,516,105
Total length of query sequence (bp)	1,921,419	1,836,297	1,788,723	1,700,858	1,737,623
Percentage of aligned query sequence region in whole genome (%)	79.57	83.75	90.3	94.03	87.25
Number of alignment block	12	18	17	4	8

Statistical analysis of single-nucleotide variations (SNVs) and InDels in *Lactobacillus salivarius* Y20 revealed that the number of insertion and deletion sites between Y20 and 2D was lower compared to those between Y20 and *Lactobacillus salivarius* strains from chicken, pig, human, and fish sources (Table 13).

**Table 13:** Statistics of SNP/InDel.

Strain name	SNP count statistics	Insertion	Deletion
Lsa_IBB3154	29,845	334	418
Lsa_JCM1046	25,888	338	334
Lsa_LPM01	25,547	310	351
Lsa_2D	22,524	287	276
Lsa_S01	25,323	467	493

When it came to the number of long-fragment sequences (≥50 bp) on the *Lactobacillus salivarius* Y20 genome, it was higher compared to IBB3154, JCM106, and LPM106, similar to S01, and lower compared to 2D. Specifically, IBB3154 had very few insertion, deletion, inversion, and translocation sites with Y20, mostly translocation sites. JCM106 had more insertion, deletion, inversion, and translocation sites with Y20 compared to IBB3154, mostly translocation and duplication sites. 2D had few insertion, deletion, and translocation sites with Y20, mostly inversion sites, with regions ranging from 0.4–1.4 Mbp. LPM01 had more insertion, deletion, inversion, and translocation sites with Y20 compared to IBB3154 and JCM106, mostly translocation, inversion, and duplication regions, with significant overlap between duplication and translocation regions in the 0.8–1.0 Mbp range. S01 only had duplication, translocation, and inversion sites with Y20 (Figure 14B).

**Figure 15:**
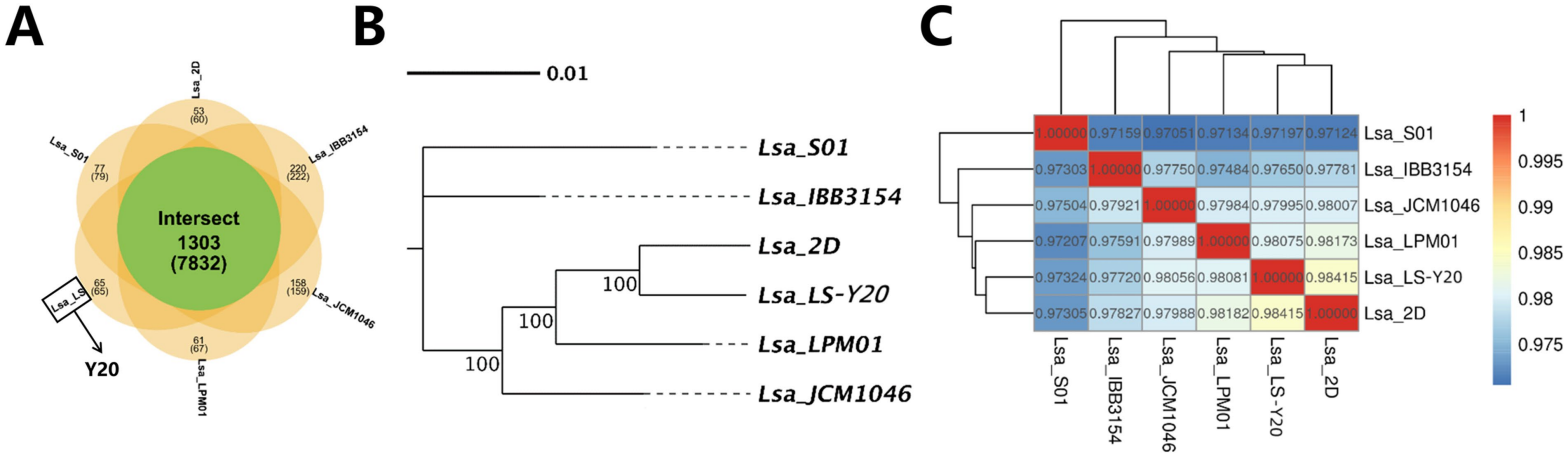
Outcomes from population evolutionary analysis. **(A)** Wayne diagram of homologous gene family. Each ellipse represents a genome. The number displayed above each region indicates the count of gene families within the species corresponding to this region. The number within parentheses below each region denotes the total number of genes across all gene families in the species of this region. **(B)** Species evolution tree. The phylogenetic tree illustrates the evolutionary relationships and topological structure among different species. At the terminal ends of the tree diagram lie the individual species, while the nodes (branching points) represent their common ancestors. The branch lengths depict the relative genetic distances between species. Numbers displayed on the branches indicate the bootstrap support values (branch confidence scores), with values closer to 100 signifying higher confidence. **(C)** Thermal diagram of ANI analysis.

Gene family analysis, which helps understand the correlation between unique genes and strain-specific traits, revealed 1,303 core genes. Y20 had 65 unique genes, IBB3154 had 220, JCM1046 had 158, LPM01 had 61, 2D had 53, and S01 had 77 (Figure 15A).

By analyzing the gene commonality between *Lactobacillus salivarius* Y20 and five other *Lactobacillus salivarius* strains, we also examined immune-related unique and core genes (Table 14). Among them, there was one immune-related unique gene, LRS64_05055 (RAMP protein csm5), which is involved in antiviral immune defense and may also participate in certain cellular autophagy mechanisms, protein synthesis regulation, and post-transcriptional modifications. Four immune-related genes were common to all six strains: LRS64_01800 (thioredoxin trxA), LRS64_03595 (thioredoxin trxA), LRS64_05635 (GTPase), and (bacteriocin immunity protein mesI). Thioredoxin trxA plays a crucial reductive role in cells, primarily participating in intracellular redox reactions. mesI is considered a pleiotropic immunity protein that can resist external bacterial invasions while also regulating internal bacterial gene expression to adapt to different environmental stresses.

**Table 14:** Immunological related gene proportion map.

Immune gene	Strain					
	Y20	IBB3154	JCM1046	LPM01	2D	S01
trxA*	+	+	+	+	+	+
mesI*	+	+	+	+	+	+
trxA*	+	+	+	+	+	+
Cas2	+	+	+		+	+
Cas1	+	+	+			+
RAMP protein Csm5#	+					
Csm5	+		+			
Csm4	+		+			
Csm3	+		+			
Csm2	+		+			
Csm1		+				
Cas6	+					
GTPase*	+	+	+	+	+	+
Cas2	+				+	
casC		+				
amtB	+	+	+	+	+	+

Genes with a * suffix after their names are common immune genes shared by the six strains. Genes with a # suffix are unique immune genes of strain Y20. A “+” indicates that the strain possesses the corresponding immune gene.

Additionally, Y20 and 2D shared two immune-related genes: LRS64_06545 (CRISPR-associated protein Cas2) and LRS64_06565 (CasC protein). The SNVs of Cas2 and CasC are listed in Tables 15, 16. Cas2 protein is important in DNA repair and genome stability. The CRISPR-Cas system is a natural immune system that resists the invasion of foreign genetic sequences.

**Table 15:** Immune shared gene SNP (Cas2) of Y20 and 2D.

Reference base	Mutant base	Mutation type	Location type
G	A	SNP	Exonic
G	A	SNP	Exonic
G	A	SNP	Exonic
T	C	SNP	Exonic
C	T	SNP	Exonic
G	A	SNP	Exonic
A	G	SNP	Exonic
C	T	SNP	Exonic

**Table 16:** Immune co existing gene SNP (CasC) of Y20 and 2D.

Reference base	Mutant base	Mutation type	Location type
A	G	SNP	Exonic
A	G	SNP	Exonic
A	G	SNP	Exonic
T	G	SNP	Exonic
A	G	SNP	Exonic
G	A	SNP	Exonic
A	G	SNP	Exonic
T	C	SNP	Exonic

Sequence alignment and phylogenetic tree construction showed that *Lactobacillus salivarius* Y20 and 2D were on the same branch with a high branch confidence value of 100. Y20, 2D, and LPM01 were on the same branch, and Y20, 2D, LPM01, and JCM1046 were on the same branch. IBB3145 and S01 had a close phylogenetic relationship but were distant from Y20, 2D, LPM01, and JCM1046 (Figure 15B).

The average nucleotide identity (ANI) values between the alignment regions of *Lactobacillus salivarius* Y20 and the reference genomes showed a high degree of discrimination among closely related species. Typically, an ANI value of 95% is used as a taxonomic threshold to distinguish different species. The results indicated that Y20 was most closely related to 2D and LPM01, with S01 being the most distant, consistent with the phylogenetic tree in 5.5.3 (Figure 15C).

## 4 Discussion

### 4.1 Isolation, screening and biological characterization analysis of vaginal lactic acid bacteria from Mongolian horses

The equine industry, while experiencing significant growth, concurrently confronts numerous reproductive health challenges, with bacterial vaginosis (BV) in mares emerging as a critical disease that severely compromises their reproductive health. This condition markedly reduces conception and fertility rates, imposing substantial economic losses on production and breeding efficiency within the equine sector and thus posing a significant bottleneck to its further industrial transformation, upgrading, and sustainable development (Coudray and Madhivanan, 2020; Marnach et al., 2022).

In traditional livestock production practices, antibiotic therapy remains the predominant approach for the treatment of bacterial vaginosis in mares. However, the prolonged and widespread use of antibiotics has triggered a series of severe problems. Antimicrobial resistance (AMR) has escalated dramatically, with many pathogens, such as *Staphylococcus aureus*, progressively developing resistance to multiple antibiotic agents and evolving into concerning “superbugs” (Liu et al., 2021; Mlynarczyk-Bonikowska et al., 2022). *S. aureus* colonizes not only the host’s gastrointestinal tract, skin, and perineal regions but also secretes diverse toxins, including alpha-hemolysin (Hla), enterotoxins, and leukocidins, which collectively endanger host health. The emergence of resistance undermines the efficacy of conventional antibiotic treatments, thereby increasing the complexity and cost of disease management (references omitted for brevity). Beyond vaginal infections, *Escherichia coli* is capable of cause severe systemic illnesses, such as diarrhea, sepsis, hemolytic uremic syndrome, and urinary tract infections, posing threats to both animal and human health (Ahmad-Mansour et al., 2021). *Salmonella* spp., as zoonotic pathogens, present substantial risks in livestock production and public health, with outbreaks potentially leading to large-scale infections in animals and humans, severely impacting the economic viability of breeding operations and public health safety (Takaya et al., 2020). *Streptococcus pyogenes* can induce severe invasive diseases, damaging local tissues and causing conditions such as scarlet fever and erysipelas, with significant implications for animal welfare (Yu et al., 2023). Although primarily a commensal bacterium in horses, *Streptococcus equi* subsp. *zooepidemicus* (SEZ) is a common pathogen in bacterial endometritis in mares and can also infect dogs, pigs, and other animals, particularly in high-value breeding sectors like horse racing, resulting in substantial economic losses (Ikhuoso et al., 2020; Boyle, 2023). *Gardnerella vaginalis* is widely implicated in bacterial vaginosis and associated with adverse reproductive health outcomes, adversely affecting mare reproductive function (Joseph et al., 2024). The AMR, drug residues, and complex pathogenicity mechanisms induced by these pathogens have emerged as critical global health threats, necessitating urgent exploration of safe, efficient, and sustainable alternative therapeutic strategies (McDonald and Linde, 2002).

Probiotics, as potential alternatives to antibiotics, have garnered considerable research attention due to their safety, controllability, eco-friendliness, and sustainability (Mnisi et al., 2025). Probiotics exhibit immense potential in modulating microecological balance, enhancing host immunity, and inhibiting pathogenic growth, offering novel solutions to combat antibiotic misuse (Suez et al., 2019; Ahire et al., 2023). Through a rigorous screening strategy, this study successfully isolated *Lactobacillus salivarius* Y20, a strain characterized by robust growth performance, strong acid tolerance, and high acid production efficiency. In preliminary screening, Y20 demonstrated rapid growth kinetics and achieved high cell density. Subsequent acid tolerance and acid production assays confirmed its ability to thrive under acidic conditions while efficiently producing organic acids. Critically, Y20 exhibited potent inhibitory effects against multiple pathogens, including *S. aureus*, *E. coli*, *Salmonella enterica* serovar Typhi, *G. vaginalis*, SEZ, and *S. pyogenes*. Theoretically, Y20’s superior growth traits enable rapid colonization and proliferation within the mare’s vaginal microenvironment, establishing dominant microbiota. Its acid tolerance ensures survival and sustained functionality in the acidic vaginal milieu, while its high acid production capacity further lowers vaginal pH, reinforcing pathogen suppression. Collectively, these attributes underscore the potential of Y20 as a probiotic for preventing and treating bacterial vaginosis in mares, offering a viable alternative to antibiotic therapy and mitigating the risks associated with AMR and drug residues. Future studies should focus on elucidating Y20’s colonization dynamics in the mare’s vagina, its long-term impact on vaginal microecology, practical therapeutic efficacy, and safety profiles. Such investigations will provide a robust theoretical foundation and empirical support for its clinical application in the equine industry, advancing the sector toward healthier, more sustainable practices.

### 4.2 Genomic characterization and functional dissection of *Lactobacillus salivarius* Y20

Whole-genome high-throughput sequencing technology has been widely employed in microbiology-related research. For the genomic study of *Lactobacillus salivarius*, the first published genome of *Lactobacillus salivarius* was that of *Lactobacillus salivarius* UCC118 (Raftis et al., 2014). The genome of *Lactobacillus salivarius* UCC118 comprises a 1.83 Mb chromosome, a large plasmid of 242 kb (pMP118), and two smaller plasmids of 20 kb (pSF118-20) and 44 kb (pSF118-44) (Fang et al., 2008). With the advancement of sequencing technology, related research has continued to increase. For instance, Ayala et al. (2017) isolated *Lactobacillus salivarius* L28 from ground beef. The genome size of L28 is approximately 2,028,405 bp, with an average GC content of 32.7%. A total of 1982 coding sequences, 64 tRNAs, and 31 rRNAs were predicted. Kergourlay et al. (2012) isolated *Lactobacillus salivarius* SMXD51 from chickens. Using the RAST server, they achieved functional annotation of predicted genes. SMXD51 contains 1795 coding sequences, 103 RNAs, and 78 tRNA genes. Raftis et al. (2014) isolated *Lactobacillus salivarius* JCM1046 from pigs, and its genome comprises a 1.83 Mb chromosome and four plasmids. Mackenzie et al. (2014) isolated *Lactobacillus salivarius* cp400 from pigs and performed automated gene prediction using GeneMark and Glimmer3. Cho et al. (2011) isolated *Lactobacillus salivarius* GJ-24 from human feces. By utilizing the RAST server and comparing with the KEGG and COG databases, they achieved functional annotation of predicted genes. Sun et al. (2015) isolated *Lactobacillus salivarius* LsR from the feces of a centenarian. Its genome contains a 1,751,565 bp circular chromosome and two plasmids. This study also identified several important genes that enable LsR to survive in the gastrointestinal tract and adhere to the intestine. Additionally, it was found that LsR can inhibit the occurrence of oral cancer and colorectal cancer caused by some factors. Jiménez et al. (2010) determined the complete genome sequence of *Lactobacillus salivarius* CECT 5713 isolated from a mother-infant pair (breast milk and infant feces) using whole-genome shotgun sequencing. Its genome consists of a 1,828,169 bp circular chromosome and two plasmids. They observed that CECT 5713 has anti-inflammatory, immunomodulatory, and anti-infective properties. In this study, the circular genome size of *Lactobacillus salivarius* Y20 is approximately 1.74 Mb, which is smaller than that of *Lactobacillus salivarius* IBB3154 (from hens), JCM1046 (from pigs), LPM01 (from humans), 2D (from horses), and S01 (from fish). Lee et al. (2017) found that the genome size of human-derived *Lactobacillus salivarius* strains is smaller than that of pig-derived strains. This could be attributed to the internal environment of humans is more attractive to strains than that of pigs and chickens, and humans are exposed to harsh environments (such as diseases, toxins, etc.) less frequently than livestock. Symbiotic bacteria accompany the evolution of the host, and in non-harsh environments, symbiotic bacteria lose more genes than in harsh environments. The genome size of *Lactobacillus salivarius* Y20 derived from the horse vagina is much smaller than that of many other sources of *Lactobacillus salivarius*. It is hypothesized that the vaginal environment of Mongolian horses is more attractive to bacteria, and the strains are exposed to fewer harsh conditions in the Mongolian horse vaginal environment.

In the analysis of the GO annotation of the Y20 genome, several antioxidant-related genes were found, such as thioredoxin, which, together with nicotinamide adenine dinucleotide phosphate and thioredoxin reductase, plays a positive role in stabilizing the redox state of organisms (Ball et al., 2022; Bradford et al., 2024). In the KEGG annotation, immune-related pathways (NOD-like receptor signaling pathway) and the TrxA gene for regulating redox were identified. Comparative genomics analysis revealed the unique genes of *Lactobacillus salivarius* Y20. One immune-related unique gene, LRS64_05055 (RAMP protein Csm5), possesses an immune defense function. Three immune-related genes are shared among six strains, namely LRS64_01800 (thioredoxin TrxA), LRS64_03595 (thioredoxin TrxA), and LRS64_05635 (GTPase). The aforementioned results collectively indicate that Y20 possesses a robust foundation for immunity and antioxidant properties.

Based on the findings of this study, several avenues for future research emerge. First, *in vivo* studies are warranted to evaluate the efficacy of Y20 in preventing or treating equine reproductive disorders. These studies should investigate the optimal dosage, duration of treatment, and potential interactions with other vaginal microorganisms. Second, further exploration of the molecular mechanisms underlying the probiotic effects of Y20, such as the production of antimicrobial substances and modulation of the host immune response, would provide deeper insights into its therapeutic potential. Additionally, comparative studies with other *Lactobacillus salivarius* strains from different hosts could identify strain-specific traits that contribute to their ecological success in specific niches. Finally, long-term safety studies are essential to ensure the absence of any adverse effects associated with the prolonged use of Y20 as a probiotic.

## 5 Conclusion

This study successfully isolated and identified a novel strain of *Lactobacillus salivarius* (Y20) from the vaginal microbiota of healthy Mongolian mares. The probiotic potential of Y20 was evaluated through a series of *in vitro* assays, demonstrating robust tolerance to low pH and bile salts, potent antagonistic activity against equine pathogens, and significant antioxidant capacity. Whole-genome sequencing and comparative genomics revealed that Y20 shares a close phylogenetic relationship with horse-derived *Lactobacillus salivarius* strains and possesses unique genomic adaptations for vaginal colonization. These findings identify *Lactobacillus salivarius* Y20 as a candidate probiotic for mitigating equine reproductive disorders, offering a sustainable alternative to antibiotics and advancing microbiome-based strategies for equine health management.

## Data Availability

The original contributions presented in the study are included in the article/Supplementary material, further inquiries can be directed to the corresponding author.

## Footnotes

^1^ www.ncbi.nlm.nih.gov
