# Supplementary material for: Isolation, identification and comparative genomic analysis of Lactobacillus salivarius from Mongolian horse vagina
Source: Front Microbiol. 2025 Jul 31;16:1635639. doi: 10.3389/fmicb.2025.1635639 (PMC12350343; doi:10.3389/fmicb.2025.1635639)
Supplement: Supplementary file 1 [file Supplementary_file_1.docx]

Supplementary Material

**Supplementary Table 1.** Statistical table of Y20 whole genome assembly results.

| **Sample name** | **Number of scaffolds** | **Genome size (bp)** | **Number of ambiguous bases (N)** | **GC content (%)** |
| --- | --- | --- | --- | --- |
| Y20 | 1 | 1,735,042 | 0 | 33.01% |

**Supplementary Table 2.** 16S small regulatory RNA (sRNA) gene sequence of *Lactobacillus salivarius* Y20.

| **Strain** | **Gene sequence** |
| --- | --- |
| *Lactobacillus salivarius* Y20 | GTTACCCCACCGGCTTTGGGTGTTACAAACTCTCATGGTGTGACGGGCGGTGTGTACAAGGCCCGGGAACGTATTCACCGCGACATGCTGATTCGCGATTACTAGCGATTCCGACTTCATGTAGGCGAGTTGCAGCCTACAATCCGAACTGAGAACGGCTTTAAGAGATTAGCTAAACCTCGCGGTCTCGCGACTCGTTGTACCGTCCATTGTAGCACGTGTGTAGCCCAGGTCATAAGGGGCATGATGACTTGACGTCGTCCCCACCTTCCTCCGGTTTGTCACCGGCAGTCTCGCCAGAGTGCCCAACTTAATGCTGGCAACTGACAACAAGGGTTGCGCTCGTTGCGGGACTTAACCCAACATCTCACGACACGAGCTGACGACAGCCATGCACCACCTGTCACTTTGTCCCCGAAGGGAAAGCCTAATCTCTTAGGTGGTCAAAGGATGTCAAGACCTGGTAAGGTTCTTCGCGTTGCTTCGAATTAAACCACATGCTCCACCGCTTGTGCGGGCCCCCGTCAATTCCTTTGAGTTTCAACCTTGCGGTCGTACTCCCCAGGCGGAATGCTTATTGCGTTAGCTGCGGCACTGAAGGGCGGAAACCCTCCAACACCTAGCATTCATCGTTTACGGCGTGGACTACCAGGGTATCTAATCCTGTTTGCTACCCACGCTTTCGAACCTCAGCGTCAGTTACAGACCAGAGAGCCGCTTTCGCCACTGGTGTTCTTCCATATATCTACGCATTTCACCGCTACACATGGAGTTCCACTCTCCTCTTCTGCACTCAAGTCTTCCAGTTTCCAATGCACTACTCCGGTTAAGCCGAAGGCTTTCACATCAGACTTAAAAGACCGCCTGCGTTCCCTTTACGCCCAATAAATCCGGACAACGCTTGCCACCTACGTATTACCGCGGCTGCTGGCACGTAGTTAGCCGTGACTTGCTGGTTAGATACCGTCATCGAATGAACAGTTACTCTCACTCGTGTTCTTCTCTAACAACAGAGTTTTACGATCCGAAGACCTTCTTCACTCACGCGGCGTTGCTCCATCAGACTTGCGTCCATTGTGGAAGATTCCCTACTGCTGCCTCCCGTAGGAGTTTGGGCCGTGTCTCAGTCCCAATGTGGCCGATCAACCTCTCAGTTCGGCTACGTATCATCACCTTGGTAGGCCGTTACCCCACCAACTAGTTAATACGCCGCGGGTCCATCTAAAAGCGATAGCAGAACCATCTTTCATCTAAGGATCATGCGATCCTTAGAGATATACGGTATTAGCACCTGTTTCCAAGTGTTATCCCCTTCTTTTAGGCAGGTTACCCACGTGTTACTCACCCGTCCGCCACTCAACTTCTTACGGTGAATGCAAGCATTCGGTGTAAGAAAGTTTCGTTCGAC |


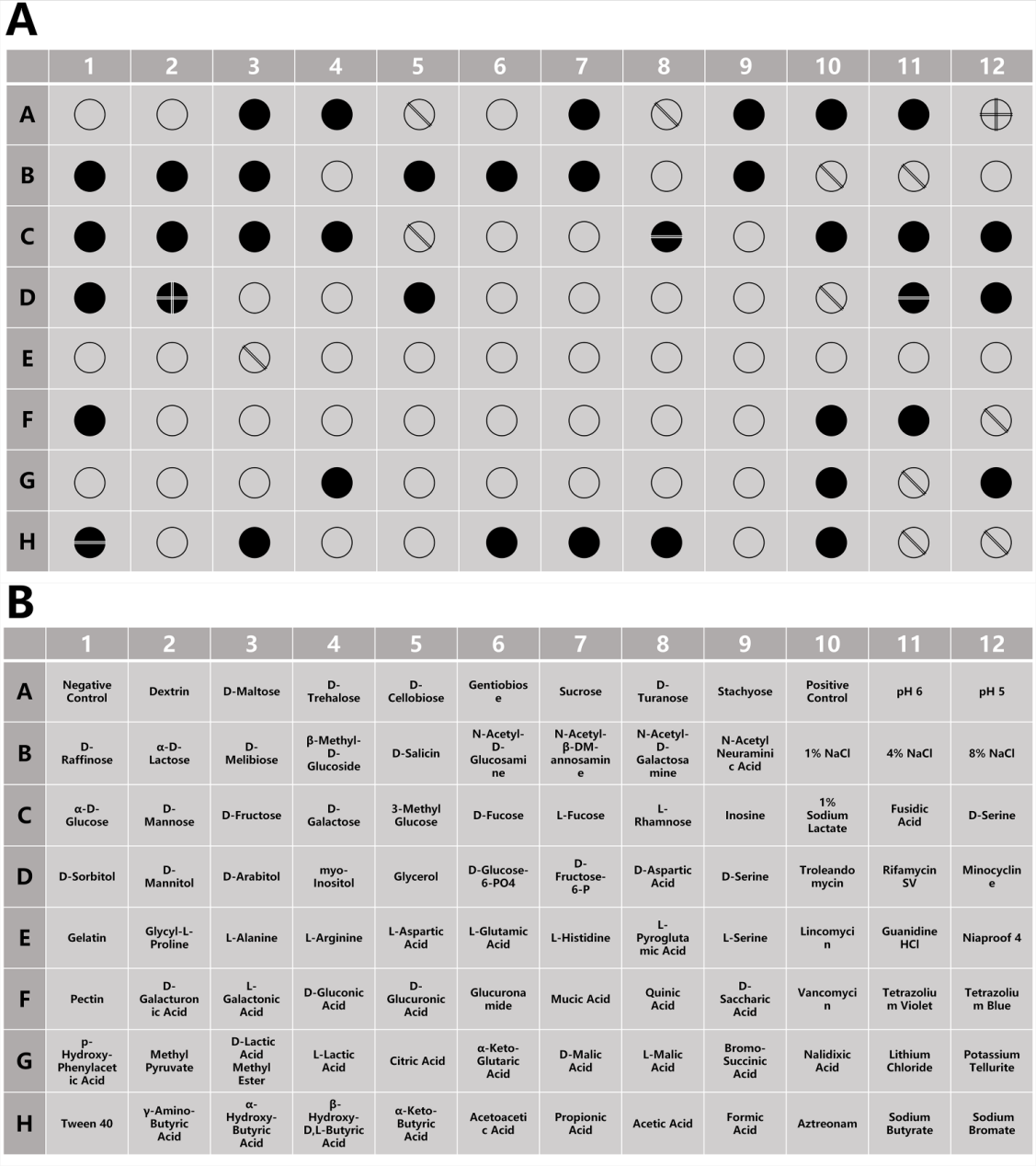


**Supplementary Figure 1.** The GEN III identification results of Y20. (A) Identification result chart of Y20 using the Biolog microplate. (B) Name of the substrate represented by each well of the microplate.


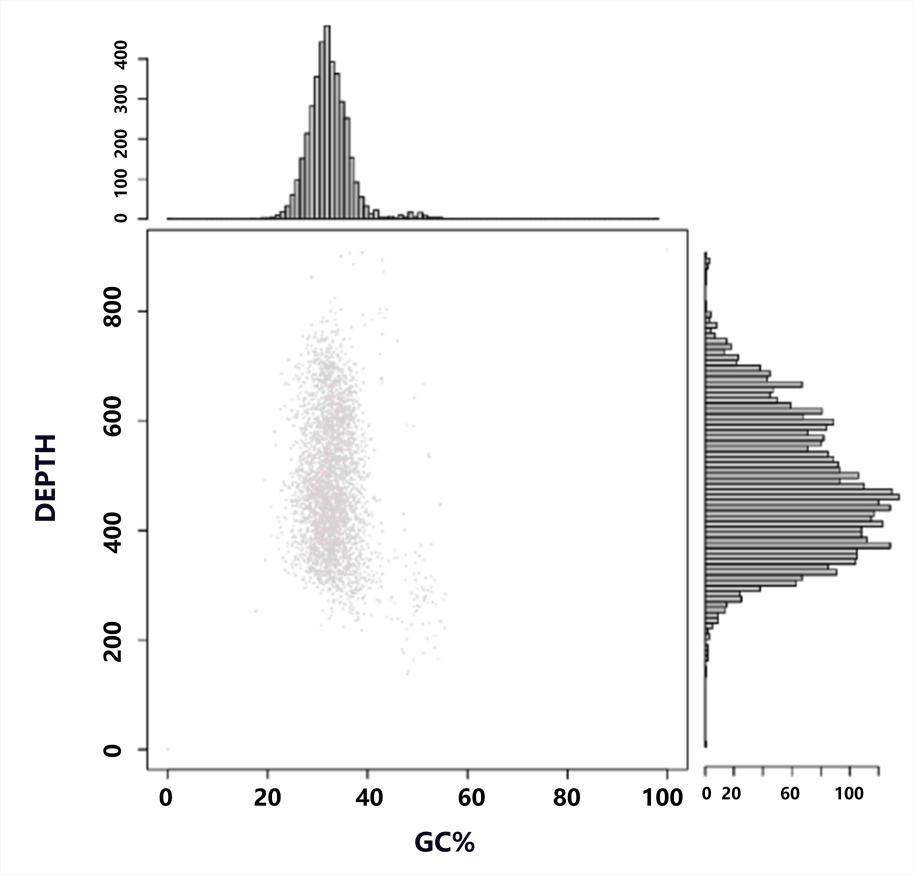


**Supplementary Figure 2.** GC-Depth distribution map.
